# Global update on the susceptibility of human influenza viruses to neuraminidase inhibitors, 2012–2013

**DOI:** 10.1016/j.antiviral.2014.07.001

**Published:** 2014-07-17

**Authors:** Adam Meijer, Helena Rebelo-de-Andrade, Vanessa Correia, Terry Besselaar, Renu Drager-Dayal, Alicia Fry, Vicky Gregory, Larisa Gubareva, Tsutomu Kageyama, Angie Lackenby, Janice Lo, Takato Odagiri, Dmitriy Pereyaslov, Marilda M. Siqueira, Emi Takashita, Masato Tashiro, Dayan Wang, Sun Wong, Wenqing Zhang, Rod S. Daniels, Aeron C. Hurt

**Affiliations:** a National Institute for Public Health and the Environment, PO Box 1, 3720 BA Bilthoven, The Netherlands; b Instituto Nacional de Saúde, Av. Padre Cruz, 1649-016 Lisboa, Portugal; c Faculdade de Farmácia, Universidade de Lisboa, Portugal; d Global Influenza Programme, World Health Organization, Avenue Appia 20, 1211 Geneva 27, Switzerland; e World Health Organization Collaborating Centre for the Surveillance, Epidemiology and Control of Influenza, Centers for Disease Control and Prevention, 1600 Clifton RD NE, MS-G16 Atlanta, GA, United States; f World Health Organization Collaborating Centre for Reference and Research on Influenza, MRC-National Institute for Medical Research, The Ridgeway, Mill Hill, London NW7 1AA, United Kingdom; g World Health Organization Collaborating Centre for Reference and Research on Influenza, National Institute of Infectious Diseases, Gakuen 4-7-1, Musashimurayama, Tokyo 208-0011, Japan; h Public Health England Colindale, 61 Colindale Avenue, London NW9 5EQ, United Kingdom; i Public Health Laboratory Centre, 382 Nam Cheong Street, Shek Kip Mei, Kowloon, Hong Kong, China; j Division of Communicable Diseases, Health Security, & Environment, World Health Organization Regional Office for Europe, UN City, Marmorvej 51, DK-2100 Copenhagen Ø, Denmark; k Respiratory Viruses Laboratory/IOC, FIOCRUZ Av Brasil, 4365 Rio de Janeiro, Brazil; l World Health Organization Collaborating Centre for Reference and Research on Influenza, Chinese National Influenza Center, National Institute for Viral Disease Control and Prevention, Chinese Center for Disease Control and Prevention, 155 Changbai Road, Changping District, Beijing 102206, China; m World Health Organization Collaborating Centre for Reference and Research on Influenza, VIDRL, At the Peter Doherty Institute for Infection and Immunity, Melbourne, VIC 3000, Australia; n University of Melbourne, Melbourne School of Population and Global Health, Melbourne, VIC 3010, Australia

**Keywords:** Influenza virus, Antiviral resistance, Neuraminidase inhibitors, Oseltamivir, Global analysis, Normalization using fold-change data

## Abstract

Emergence of influenza viruses with reduced susceptibility to neuraminidase inhibitors (NAIs) is sporadic, often follows exposure to NAIs, but occasionally occurs in the absence of NAI pressure. The emergence and global spread in 2007/2008 of A(H1N1) influenza viruses showing clinical resistance to oseltamivir due to neuraminidase (NA) H275Y substitution, in the absence of drug pressure, warrants continued vigilance and monitoring for similar viruses. Four World Health Organization (WHO) Collaborating Centres for Reference and Research on Influenza and one WHO Collaborating Centre for the Surveillance, Epidemiology and Control of Influenza (WHO CCs) tested 11,387 viruses collected by WHO-recognized National Influenza Centres (NIC) between May 2012 and May 2013 to determine 50% inhibitory concentration (IC_50_) data for oseltamivir, zanamivir, peramivir and laninamivir. The data were evaluated using normalized IC_50_ fold-changes rather than raw IC_50_ data. Nearly 90% of the 11,387 viruses were from three WHO regions: Western Pacific, the Americas and Europe. Only 0.2% (*n* = 27) showed highly reduced inhibition (HRI) against at least one of the four NAIs, usually oseltamivir, while 0.3% (*n* = 39) showed reduced inhibition (RI). NA sequence data, available from the WHO CCs and from sequence databases (*n* = 3661), were screened for amino acid substitutions associated with reduced NAI susceptibility. Those showing HRI were A(H1N1)pdm09 with NA H275Y (*n* = 18), A(H3N2) with NA E119V (*n* = 3) or NA R292K (*n* = 1) and B/Victoria-lineage with NA H273Y (*n* = 2); amino acid position numbering is A subtype and B type specific. Overall, approximately 99% of circulating viruses tested during the 2012–2013 period were sensitive to all four NAIs. Consequently, these drugs remain an appropriate choice for the treatment and prophylaxis of influenza virus infections.

## 1. Introduction

Neuraminidase inhibitors (NAIs) are the most widely used antiviral drugs for the treatment or prophylaxis of influenza. The older class of influenza antivirals, the adamantanes, are currently not recommended for use due to the high frequency (>99%) of resistance in circulating influenza A viruses [A(H1N1)pdm09 and A(H3N2)] and their ineffectiveness against influenza B viruses (Simonsen et al., 2007; Nelson et al., 2009; Tisdale, 2009; Gubareva et al., 2010). The NAIs oseltamivir and zanamivir have been approved for use in many countries since 1999/2000, while two other NAIs, peramivir and laninamivir, have to date been approved in Japan, and in the case of peramivir also in the Republic of Korea and China.

The NAIs bind to the neuraminidase (NA) glycoprotein on the surface of influenza A and B viruses, restricting the capacity of these viruses to release from host cells, a critical stage of virus replication. Substitutions of amino acids located in or close to the NA active site can lead to reductions in NAI binding and effectiveness of drug treatment. Such viruses are typically described as being ‘resistant’ to or showing ‘reduced inhibition’ or ‘highly reduced inhibition’ by particular NAIs; these terms might be confusing, as ‘resistant’ relates to clinical effectiveness and ‘reduced inhibition/highly reduced inhibition’ to the biological characteristics of the NA. Therefore clear definitions were formulated by the World Health Organization (WHO) Global Influenza Surveillance and Response System (GISRS) expert working group on surveillance of influenza antiviral susceptibility (WHO-AVWG) using 50% inhibitory concentration (IC_50_; the concentration of drug required to inhibit a standardised amount of NA activity by 50%) fold-change thresholds, compared to the mean or median for viruses from the same type/subtype/lineage showing ‘normal inhibition’ (NI), for reporting and classifying the NAI susceptibility of viruses to individual NAIs (WHO, 2012). The rationale for the selection of specific fold-change values is described in detail in a previous report (WHO, 2012). Those showing ‘reduced inhibition’ (RI) are influenza A viruses that have a 10- to 100-fold increase in IC_50_, or influenza B viruses with a 5- to 50-fold increase in IC_50_. Viruses showing ‘highly reduced inhibition’ (HRI) are influenza A viruses with a >100-fold increase in IC_50_ or influenza B viruses with a >50-fold increase in IC_50_ (WHO, 2012). Infections with viruses showing HRI are considered ‘clinically resistant’ if treatment with the antiviral drug for which the virus shows HRI has reduced clinical effectiveness.

In most years the frequency of circulating influenza viruses showing RI or HRI is less than 1%, but occasionally influenza viruses with RI or HRI spread widely within a community (Hurt et al., 2011; Garg et al., 2013). The most extreme example was in 2007/2008, when the former seasonal A(H1N1) virus acquired the NA H275Y substitution and spread globally in approximately 12 months (Lackenby et al., 2008; Collins et al., 2009; Dharan et al., 2009; García et al., 2009; Hauge et al., 2009; Hurt et al., 2009; Meijer et al., 2009). This NA substitution conferred HRI by both oseltamivir and peramivir, and was shown in clinical situations to render oseltamivir, the most widely used NAI, significantly less effective for the treatment of this virus infection (Kawai et al., 2009; Dharan et al., 2010). Amino acid substitutions D344N and R222Q in the NA of former seasonal A(H1N1) compensated for the detrimental effect of the HRI H275Y substitution on virus fitness, allowing the virus to spread efficiently (Abed et al., 2011; Rameix-Welti et al., 2011). As regional differences in NAI use exist, high use in Japan and the United States of America (USA) and low use in other parts of the world, this could potentially bias the results in a given region if post treatment reduced susceptibility is prevalent and a substantial proportion of such viruses are submitted to the WHO CC. However, to date such regional differences have not been observed.

On an annual basis, the WHO convenes a technical consultation, the WHO-AVWG, comprised of representatives from the WHO Collaborating Centres for Reference and Research on Influenza (WHO CCs), a selection of WHO-recognized National Influenza Centres (NICs) and public health institutes and research laboratories with expertise in influenza antiviral susceptibility surveillance (WHO, 2012, 2013). The meeting of the WHO-AVWG provides an opportunity to analyse the NAI susceptibility data for influenza viruses collected across 126 countries by GISRS laboratories over the previous 12 months. In this paper, which is the first of a series of annual reports, we present the results for viruses collected via GISRS and analysed by five WHO CCs between May 2012 and May 2013 (subsequently referred to as 2012–2013).

## 2. Overall analysis of phenotypic antiviral susceptibility data from WHO CCs

Five WHO CCs (Atlanta, United States; Beijing, China; London, United Kingdom; Melbourne, Australia; and Tokyo, Japan; http://www.who.int/influenza/gisrs_laboratory/collaborating_centres/list/en/). provided IC_50_ and NA amino acid substitution data for virus isolates, notably for those showing RI or HRI by NAIs, with specimen collection dates between week 21/2012 (19/5/2012) through week 20/2013 (19/5/2013) inclusive. Epidemiologic data for patient gender, age, geographic location, setting (community, hospitalised and sentinel/non-sentinel specimen collection), antiviral treatment history and immune status were also included in the analyses when available. All five WHO CCs tested for oseltamivir and zanamivir susceptibility, and additionally the Atlanta, Melbourne and Tokyo WHO CCs tested for peramivir and laninamivir susceptibility.

**Fig. 1.**
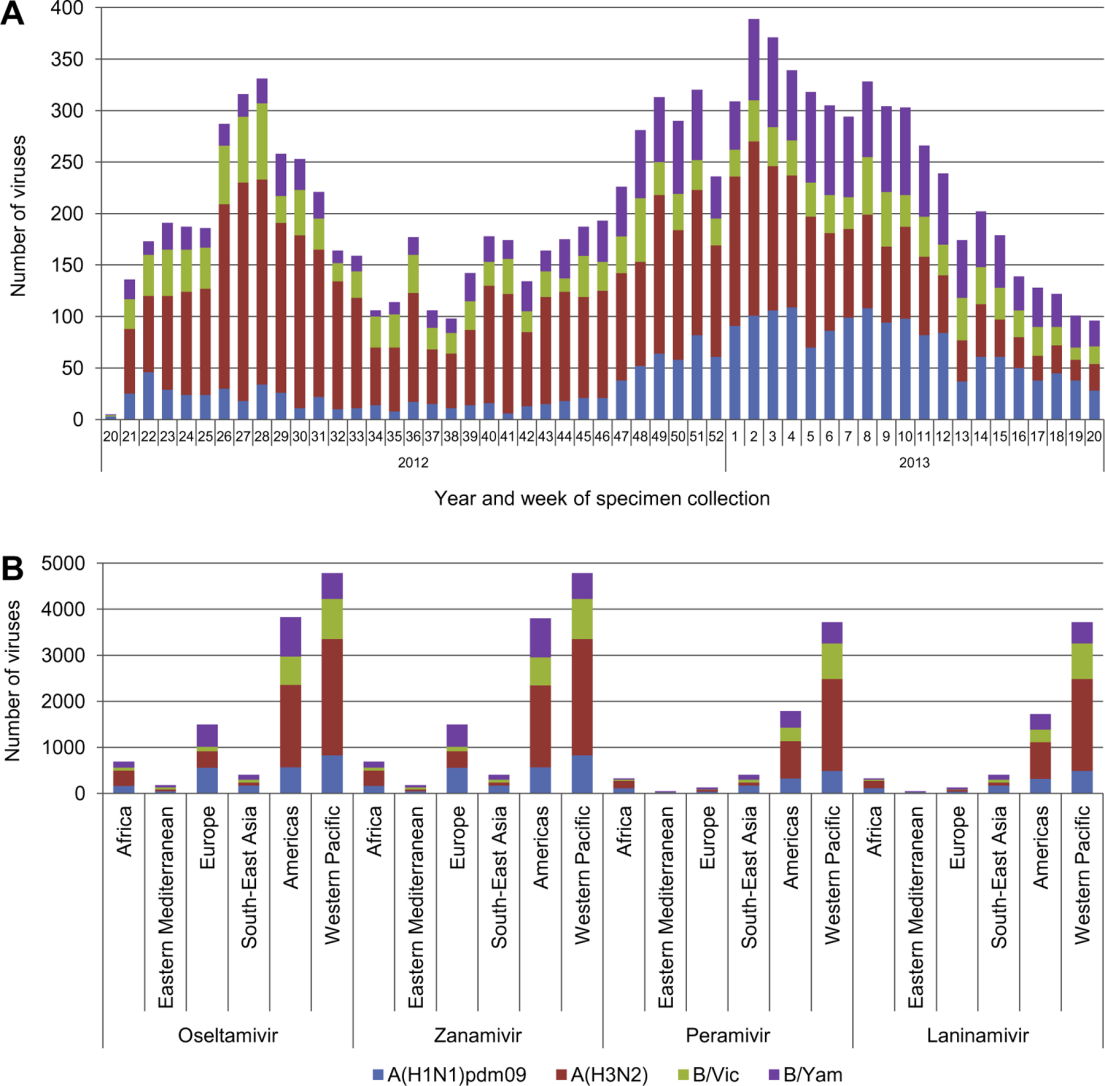
Influenza viruses collected and tested for NAI susceptibility during 2012–2013. (A) Type/subtype and specimen collection dates for specimens tested demonstrates two peaks in specimen collection, during the Southern Hemisphere winter in week 28, 2012, and the Northern Hemisphere winter in week 2, 2013. (B) Number of viruses tested for susceptibility to the four NAIs by WHO region. The greatest number of viruses tested was from the Western Pacific Region and the Americas. All viruses were tested for susceptibility to oseltamivir and zanamivir and a high proportion against peramivir and laninamivir.

The WHO CCs tested 11,387 viruses, collected during the 2012–2013 period, for NAI susceptibility using local implementations of the fluorescence-based neuraminidase inhibition assay described by Potier et al., 1979. The viruses rescued are mostly derived from community surveillance specimens collected at first encounter of the patient, so they are unlikely to have been treated with NAI. A small proportion of viruses are likely to be derived from patients during or after treatment with NAI, but the actual number is unknown as antiviral treatment information is not available for many of the specimens submitted to the WHO CCs. The number tested was well distributed across the time period but with increased numbers over the periods of epidemics in the Southern and Northern Hemispheres (Fig. 1A). Across the 12 months there were 5109 (45%) A(H3N2), 2343 (21%) A(H1N1)pdm09, 2172 (19%) B/Yamagata-lineage and 1763 (15%) B/Victoria-lineage viruses tested. By WHO region (http://www.who.int/about/structure/en/), 42% of viruses tested originated from the Western Pacific Region, 34% from the Americas, 13% from Europe, 6% from Africa, 4% from South-East Asia and 2% from the Eastern Mediterranean Region (Fig. 1B).

**Fig. 2.**
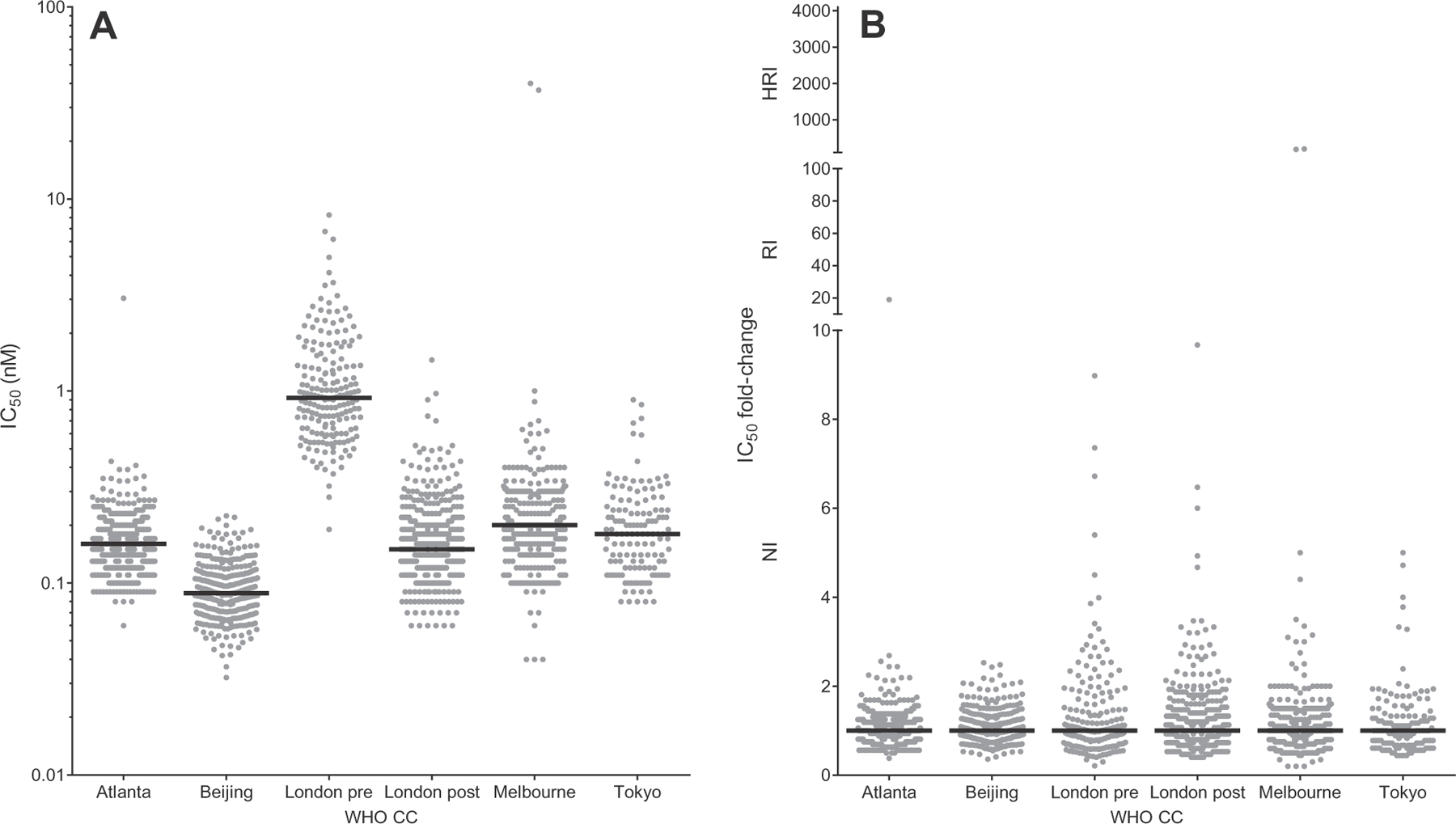
Examples for A(H1N1)pdm09 viruses and zanamivir of the variation in IC_50_ values between WHO CCs, before (A) and after normalization using IC_50_ fold-changes (B). As the London WHO CC changed its equipment (fluorometer) halfway through the study period, their data is displayed for the two periods separately; *London pre* and *London post*. Fold-changes have been calculated compared to the A(H1N1)pdm09 specific median (four WHO CCs) or mean (Tokyo WHO CC) IC_50_ values provided by the WHO CCs. The *Y*-axis of Figure B has been split into 3 compartments according to the WHO-AVWG recommended thresholds for normal inhibition (NI) (A viruses <10-fold), reduced inhibition (RI) (A viruses 10- to 100-fold), and highly reduced inhibition (HRI) (A viruses >100-fold). The median IC_50_ or IC_50_ fold-change values are indicated with a black horizontal bar.

The methodologies used for the phenotypic NA enzyme inhibition assays in the five WHO CCs are different, resulting in variation of IC_50_ values between the laboratories for a panel of reference viruses (Table 1) and test viruses (examples in Fig. 2; full details for each A subtype and B lineage by WHO CC and NAI in Supplementary Fig. 1). In addition, the London WHO CC changed its equipment halfway through the study period, so their data for the two periods were analysed separately.

**Fig. 3.**
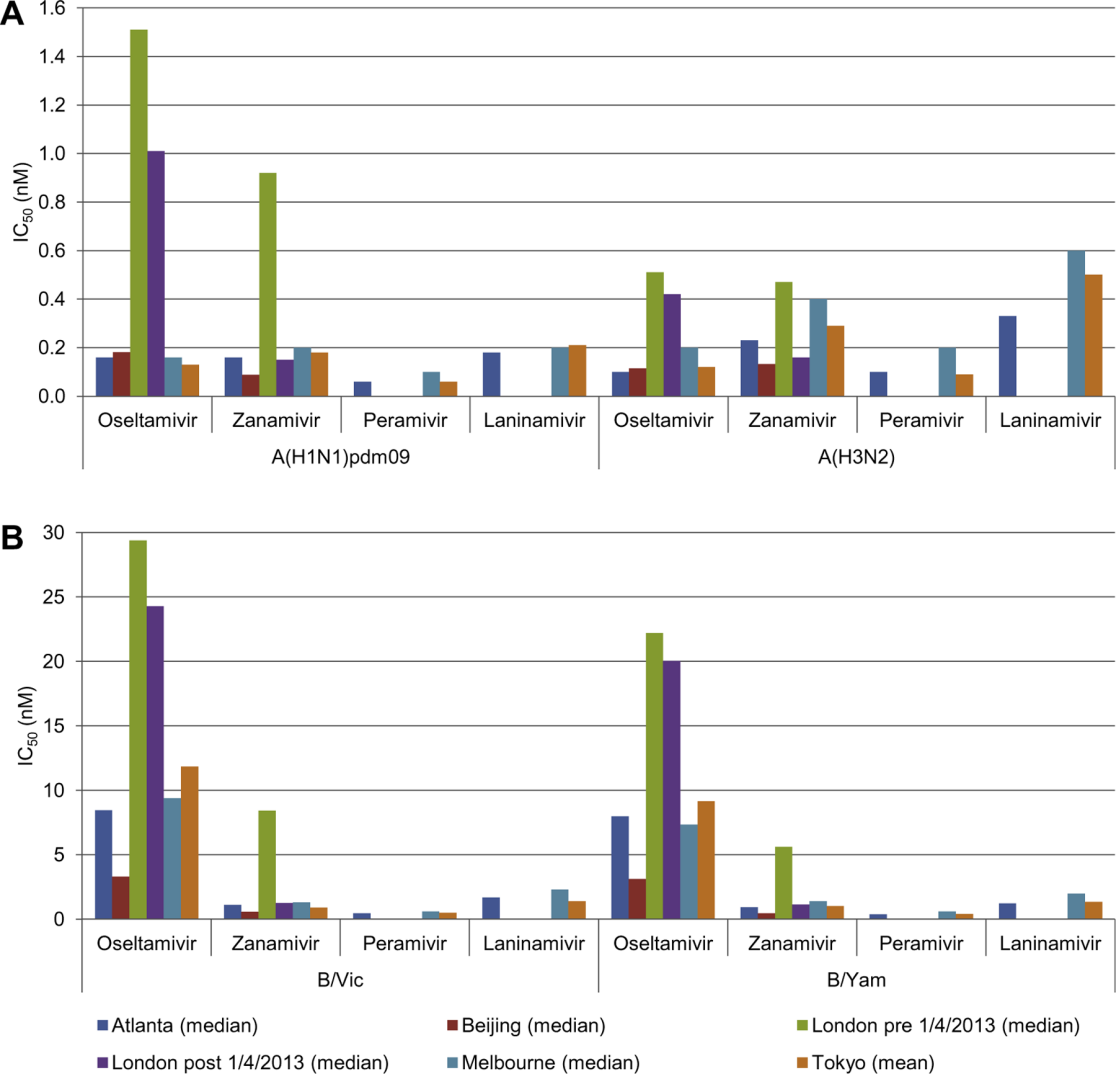
Comparison of raw IC_50_ data generated by the five WHO CCs. (A) A(H1N1)pdm09 and A(H3N2) viruses. (B) B/Victoria- and B/Yamagata-lineage viruses. Displayed are median (four WHO CCs) or mean (Tokyo WHO CC) IC_50_ values as reported by the WHO CCs. As the London WHO CC implemented a change in equipment on 01 April 2013, median IC_50_ values are displayed for the period before and after 01 April 2013. These median/mean values were used to calculate the fold-change values presented in Fig. 4.

Comparison of the median (four WHO CCs) and mean (Tokyo WHO CC) NAI-specific IC_50_ values reported by individual WHO CCs for each virus subtype or lineage revealed sufficient variation between laboratories to prevent the pooling of raw IC_50_ data (Fig. 3). All 16 datasets (4 virus subtypes or lineages and 4 NAIs) showed significant differences (*p* < 0.05) between WHO CCs by one-way ANOVA Kruskal–Wallis test of the median of log-transformed IC_50_ values (Supplementary Table 1). By pairwise comparison of the log-transformed IC_50_ values, 125 of the 144 comparisons appeared to be statistically different (Dunn’s multiple comparison test; *p* < 0.05) (Supplementary Table 1). As an alternative to using raw IC_50_ data, a fold-change in IC_50_ was determined for each test virus compared to the median (four WHO CCs) or mean (Tokyo WHO CC) NAI-specific IC_50_ values reported by individual WHO CCs for each virus subtype or lineage. By using this conversion of all raw IC_50_ data into relative fold-change values, the distributions could be normalized, thereby allowing a pooled analysis of the data from all five WHO CCs (examples in Fig. 2; full details for each A subtype and B lineage by WHO CC and NAI in Supplementary Fig. 1). By pairwise comparison, the percentage statistically different comparisons reduced from 87% (125/144) with raw IC_50_ data to 8% (11/144) with fold-change data (Supplementary Table 1). In addition, the level of significance was less strong with fold-change data (median significant *p*-value 0.0026; range <0.0001–0.0312) than with the raw IC_50_ data (median significant *p*-value <0.0001; range <0.0001–0.0461).

**Fig. 4.**
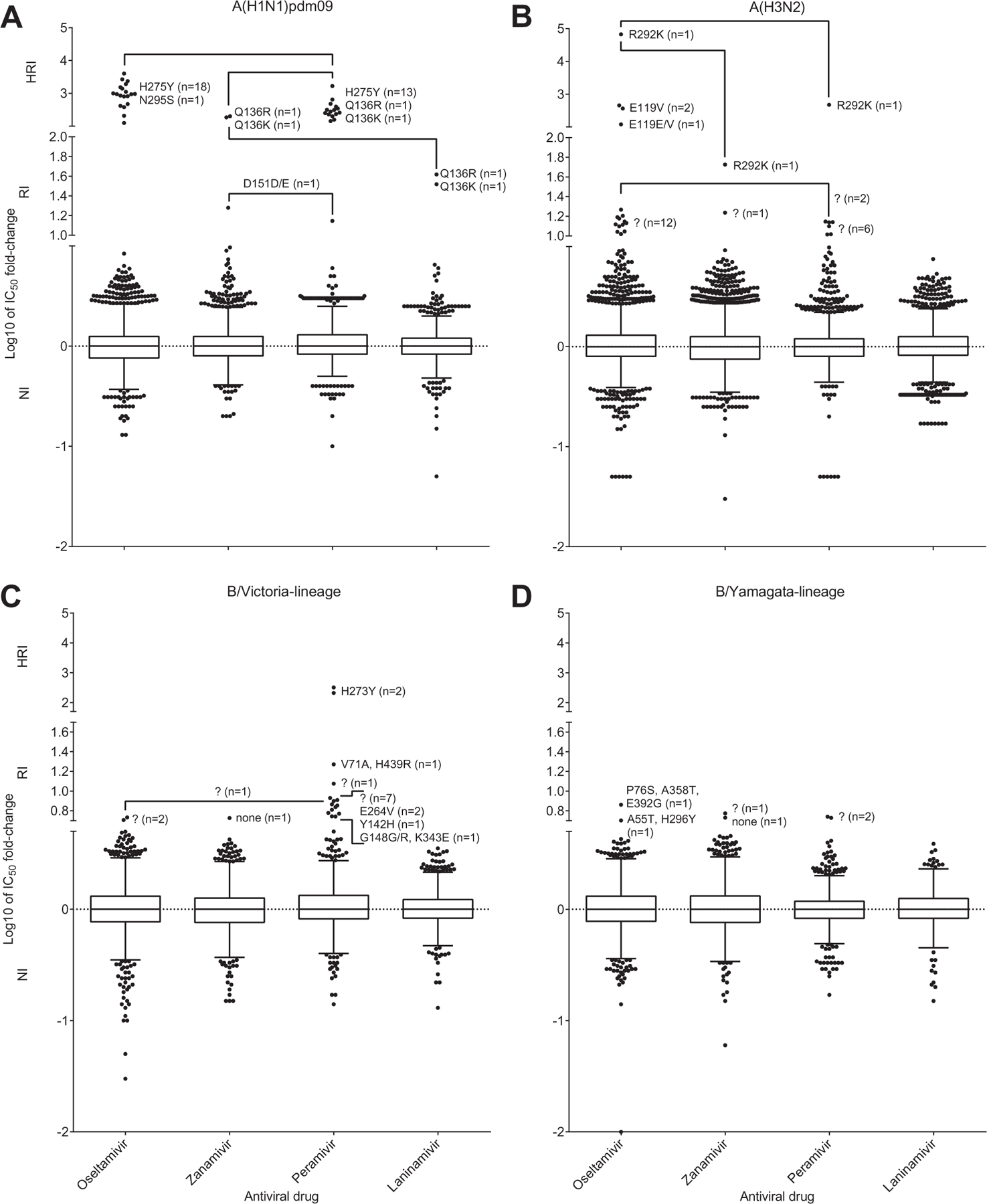
Column-scatter plots of log-transformed IC_50_ fold-change values. Data are presented by virus subtype or lineage (A) A(H1N1)pdm09; (B) A(H3N2); (C) B/Victoria-lineage; (D) B/Yamagata-lineage) and neuraminidase inhibitor (labelled on the *X*-axis: oseltamivir, zanamivir, peramivir, laninamivir). The boxes indicate the 25–75 percentile and the whiskers stretch to the lowest and highest value within 1.5 times the interquartile region value from both the 25 and 75 percentile values respectively (Tukey’s definition). The *Y*-axes have been split into 3 compartments according to the WHO-AVWG recommended thresholds for normal inhibition (NI) (A viruses <10-fold; B viruses <5-fold), reduced inhibition (RI) (A viruses 10- to 100-fold; B viruses 5- to 50-fold), and highly reduced inhibition (HRI) (A viruses >100-fold; B viruses >50-fold). For RI and HRI viruses that have been sequenced the determined amino acid substitutions are shown; amino acid position numbering is A subtype and B type specific. Connecting lines indicate viruses that have RI and HRI with more than one NAI. For RI viruses that fall close to the NI/RI intersect, the question mark (?) indicates that information on amino acid substitutions was not available.

IC_50_ fold-change graphs were constructed based on log-transformed IC_50_ fold-change data utilizing a box-and-whisker plot analysis using Tukey’s method to display the range of data and outliers (Fig. 4). Additionally, the box-and-whisker plots were constructed with the *Y*-axis split into three sections indicating the IC_50_ fold-change range for viruses classified as NI, RI or HRI based on the criteria above (Fig. 4). Among 11,387 viruses tested, 66 viruses (0.6%) showed RI or HRI to one or more of the NAIs (Fig. 4 and Table 2). The NA genes from 38 of these viruses were sequenced, with 35 of them encoding an amino-acid substitution in the NA compared to wild type viruses showing NI (Table 2).

## 3. A(H1N1)pdm09 viruses showing RI or HRI

Twenty two A(H1N1)pdm09 viruses out of 2343 tested (1%) showed RI or HRI by one or more of the NAIs (Fig. 4; Table 2). The most commonly detected NA amino acid substitution was H275Y, present in 18 viruses showing RI or HRI by oseltamivir (22 viruses tested) and peramivir (13 viruses tested). Of the 18 H275Y variants, six were from the Western Pacific, six from the Americas, five from Europe and one from South-East Asia, from 11 countries in total. The H275Y substitution conferred 207- to 4010-fold higher oseltamivir IC_50_ values and 157- to 1672-fold higher peramivir IC_50_ values compared to wild type viruses, but had little or no effect on zanamivir or laninamivir susceptibility (Table 2). Of the nine clinical specimens for which sequence results were available, all contained the H275Y substitution. Because former seasonal A(H1N1) viruses possessing NA H275Y substitution spread globally in 2008 (Lackenby et al., 2008; Collins et al., 2009; Dharan et al., 2009; García et al., 2009; Hauge et al., 2009; Hurt et al., 2009; Meijer et al., 2009), and a cluster of A(H1N1)pdm09 viruses with NA H275Y substitution was detected in untreated community cases in Australia in 2011 (Hurt et al., 2011), there has been significant concern that such variant viruses, with HRI, may acquire the ability to spread widely.

Of the H275Y variant viruses detected in 2012–2013 where patient setting and antiviral treatment information was available, 80% were from non-hospitalised patients that had not been treated with oseltamivir, suggesting that there is potential for spontaneous emergence and spread of these resistant viruses in the community. In addition, all five patients for whom immune status information was available were reported as not being immunocompromised. NA amino acid substitutions D151E, Q136K and Q136R were detected, once each, in viruses with altered NAI susceptibility, but such substitutions can emerge during MDCK cell culture (Okomo-Adhiambo et al., 2010). The clinical specimens yielding two of these isolates were unavailable, but the specimen yielding the Q136R isolate did not contain the mutation conferring the amino acid substitution prior to cell culture. As the IC_50_ data reflect the findings for virus isolates tested by the WHO CCs, these viruses were included in the total count of viruses showing RI or HRI. One A(H1N1)pdm09 virus isolate from Argentina, A/Salta/1341/2012, contained a N295S amino acid substitution which conferred HRI by oseltamivir but had only a mild effect on zanamivir susceptibility. The NA N295S substitution was subsequently confirmed in the clinical specimen from which A/Salta/1341/2012 was isolated. This NA N295S substitution has been reported previously in clinical specimens (Morlighem et al., 2011).

## 4. A(H3N2) viruses showing RI or HRI

Twenty one A(H3N2) viruses out of 5109 tested (0.4%) showed RI or HRI by one or more of the NAIs (Fig. 4; Table 2). The most commonly detected NA amino acid substitution was E119V, present in three of the five viruses for which sequences were available (Table 2), two from the Americas and one from the Western Pacific. The E119V substitution conferred increases in oseltamivir IC_50_ values ranging from 120- to 454-fold compared to that of A(H3N2) viruses showing NI, while retaining NI by zanamivir, laninamivir and peramivir. Although treatment information was unavailable for one of the cases, two of the E119V variant viruses came from patients undergoing oseltamivir treatment. An A(H3N2) virus with a R292K NA substitution was also recovered from an oseltamivir-treated patient. This virus showed a >60,000-fold increase in oseltamivir IC_50_, a 474-fold increase in peramivir IC_50_ and a 53-fold increase in zanamivir IC_50_ compared to values for A(H3N2) viruses displaying NI by the three NAIs (Table 2). The E119V and R292K substitutions were also detected in the corresponding clinical specimens. The remaining 17 A(H3N2) viruses had IC_50_ values that were around the intersect between NI and RI categories, and were either not sequenced or sequenced but with no NA amino acid substitutions being detected (Table 2).

## 5. B/Victoria-lineage viruses showing RI or HRI

Seventeen B/Victoria-lineage viruses out of 1763 tested (1%) showed RI or HRI by one or more of the NAIs (Fig. 4; Table 2); of 16 of these viruses tested against peramivir, 15 showed RI or HRI and 13 of these 15 showed NI to the other three NAIs. Two viruses from epidemiologically related cases in the Western Pacific Region contained NA H273Y substitutions which conferred 210- to 322-fold increases in peramivir IC_50_ but had no effect on susceptibility to the other NAIs. All of the isolates that were classified as having RI to peramivir require further analysis to confirm that the NA substitutions identified in the isolates are responsible for the changes in IC_50_. In two cases for which sequence results for the clinical specimens were available, the NA substitutions associated with RI or HRI were not found, further demonstrating the potential for viruses with RI or HRI to arise during cell culture (Table 2).

## 6. B/Yamagata-lineage viruses showing RI or HRI

Only six B/Yamagata-lineage viruses out of 2172 tested (0.3%) showed RI by at least one of the NAIs (Fig. 4; Table 2). All six viruses fell at the intersect between NI and RI (the greatest IC_50_ fold increase for these six viruses compared to the median was 7.3-fold) so although some NA substitutions were detected, further investigation is needed to assess the role of these substitutions in altering NAI susceptibility.

## 7. Frequency of RI and HRI conferring NA amino acid substitutions in sequence databases

On the WHO website, the AVWG lists NA amino acid substitutions that (i confer either clinical resistance/HRI (e.g. H275Y) or HRI/RI *in vitro*, and (ii have been confirmed to occur in patient clinical specimens (not just virus isolates). The list of amino acid substitutions is available at: http://www.who.int/influenza/gisrs_laboratory/antiviral_susceptibility/nai_overview/en/ (accessed 9 May 2014), and displayed in Tables 3 and 4. This list is reassessed by the AVWG on an annual basis and updated as required to include new NA substitutions that confer reduced susceptibility. For example, the list currently requires the inclusion of the B NA I221V substitution that was present in a cluster of influenza B viruses detected in North and South Carolina, United States of America, in 2011/2012 (Garg et al., 2013) and the B NA I221L substitution that emerged in an immunocompromised patient treated with oseltamivir and yielded viruses showing HRI (Escuret et al., 2014). However the merits of including substitutions such as D199N in N1 described below, that confer only a few fold increase in IC_50_, and therefore remain classified as showing NI, requires further discussion. Nevertheless, the list enables scientists to screen NA sequence data for known substitutions that have previously been shown, via functional assays, to alter (decrease) NAI susceptibility.

Using the list we screened NA sequences from viruses collected during the 2012–2013 period that had been deposited on influenza sequence databases (Global Initiative on Sharing All Influenza Data [GISAID] at www.gisaid.org and National Center for Biotechnology Information Influenza Virus Resource [NCBI-IVR] at www.ncbi.nlm.nih.gov/genomes/FLU/FLU.html).

Following the curation of sequence data to ensure short, duplicate or poor quality sequences were removed, a total of 3661 sequences from GISAID and NCBI-IVR were analysed for the presence of key NA amino acid substitutions (Supplementary Table 2). Many of the sequences in the databases were from viruses collected through the GISRS network, and therefore there was significant overlap between the viruses represented in the databases, and those characterised via phenotypic analysis in GISRS laboratories; 2826/3661 (77%) of the sequences were deposited by WHO CCs and were included in the IC_50_ dataset of the WHO CCs (Tables 3 and 4 and Supplementary Table 3). Twenty-two of the 1083 (2%) N1 sequences from A(H1N1)pdm09 viruses contained the H275Y substitution (Table 3), of which 16 had been analysed by NA inhibition assay at one of the WHO CCs and are included in the viruses shown in Fig. 4A and Table 2. However, there were six NA H275Y variant viruses from Brazil (*n* = 3), Sweden (*n* = 1), India (*n* = 1) and Lao People’s Democratic Republic (*n* = 1) that were present in the sequence databases that had not been analysed/reported within the GISRS network and therefore not included in the WHO CCs IC_50_ dataset. Including the six NA H275Y viruses reported in the sequence database only and the two NA H275Y viruses reported in the WHO CC dataset only (1 from China and 1 from Singapore), the 24 NA H275Y viruses were derived from 14 countries worldwide and specimens collected throughout the reporting period. No clear clustering was found, although the two NA H275Y viruses from the Netherlands were associated with possible transmission at a holiday location (Meijer et al., 2012) and the four NA H275Y viruses from Brazil were associated with possible community transmission in the south of the country (Souza et al., 2013). Of the 1083 N1 sequences 1054 (97%) contained V241I substitution, and 1050 (97%) N369 K substitution, which are compensatory for H275Y substitution, thereby increasing the risk of emergence of fit oseltamivir HRI A(H1N1)pdm09 viruses that could spread worldwide (Butler et al., 2014).

Analysis of the available sequences also identified two N1 sequences containing D199N substitution and one influenza B NA sequence containing D197N substitution (Table 4). The viruses with the D199N substitution showed 2- to 3-fold increases in oseltamivir IC_50_, values similar to the 3-fold increase listed in the AVWG table based on a previous study (Ghedin et al., 2011), however they were below the threshold of RI by definition. The influenza B virus containing the NA D197N substitution showed only a 2-fold increase in oseltamivir IC_50_ (therefore classified as NI), although in the AVWG table, it is listed that the substitution confers 4- to 10-fold increases in IC_50_ based on previous studies (Mishin et al., 2005; Ison et al., 2006; Hatakeyama et al., 2007; Sheu et al., 2008). The differences in IC_50_ fold-change may indicate that some amino acid substitutions have varying degrees of effect dependent on additional substitutions that may have occurred in the NA in the course of virus evolution, i.e. effects may differ dependent on the NA backbone a particular substitution occurs in. Two NA sequences identified in the databases, one with an N2 E119V substitution and the other a B NA I221T substitution (Table 4), were not among the viruses tested by phenotypic assays at the WHO CCs.

## 8. Concluding remarks

The WHO-AVWG was able to perform this global analysis on influenza antiviral susceptibility thanks to NICs within the WHO GISRS fulfilling their Terms of Reference (ToR) by collecting influenza virus positive clinical specimens and sharing a representative proportion of them, or viruses recovered, with the WHO CCs for further detailed characterization (Kitler et al., 2002). However, the WHO CC data contained low numbers of viruses from the WHO regions of Africa, South-East Asia and the Eastern Mediterranean, possibly due to the difficulties in setting up surveillance systems and establishing NICs in these regions. Pooling of IC_50_ data generated by WHO CCs was possible using methodology based on IC_50_ fold-change values, developed by the WHO-AVWG, and validated as described in this paper. This methodology could be used to include IC_50_ data generated by NICs in pooled analysis if a suitable reporting system was in place, thereby increasing the coverage and representativeness to obtain a more robust measure of global influenza antiviral susceptibility.

Based on our current analysis, approximately 99% of all viruses circulating during 2012–2013 were sensitive to all four NAIs and therefore these drugs remain an appropriate choice for the treatment and prophylaxis of influenza virus infections. Our data demonstrated that RI and HRI viruses are not circulating at significant levels in the communities providing viruses; however, surveillance data does not allow significant insight about post-treatment RI and HRI viruses. Plans of the WHO-AVWG to improve the data collection and analysis include:

1. The use of only the median of NI viruses to calculate the fold-change values to further standardise data interpretation;
2. Sequencing of the NA of all viruses showing RI or HRI, including those that are just above the threshold for RI; and
3. Definition of a minimum critical clinical and epidemiological dataset to be included with a virus isolate or clinical specimen submitted to a WHO CC.

The latter will provide an opportunity to include further information on risk factors for the emergence of viruses with reduced susceptibility, such as an immunocompromised status while being treated with NAI, prolonged therapy with NAI, and prophylaxis. However, a major hurdle in collecting this type of data that cannot be addressed by the WHO CCs, is the ability of NICs to obtain this level of information in a timely manner. Importantly, obtaining this information should not delay submission of viruses or virus positive clinical specimens to NICs and subsequently to WHO CCs for virus characterisation in relation to the scheduled WHO vaccine recommendation meetings in February and September each year.

## Supplementary Material

Supp 1

Supp 2

Supp 3

Supp 4

## Figures and Tables

**Table 1:** Antiviral susceptibility results for a panel of reference viruses^a^ of four participating WHO CCs.

Strain designation	Virus characteristics		Oseltamivir IC_50_ (nM ± 1SD) by WHO CC				Zanamivir IC50 (nM ± 1SD) by WHO CC			
	Type/subtype	NA-substitution^b^	Atlanta, United States (*n* = 3)	Tokyo, Japan (*n* = 3)	Melbourne, Australia (*n* = 3)	London, United Kingdom (*n* = 1)	Atlanta, United States (*n* = 3)	Tokyo, Japan (*n* = 3)	Melbourne, Australia (*n* = 3)	London, United Kingdom (*n* = 1)
B/Perth/211/2011	B	197D	9.5 ± 0.4	17.3 ± 3.5	19.2 ± 4.4	9.7	1.1 ± 0.2	1.3 ± 0.2	1.3 ± 0.3	0.7
B/Perth/211/2011	B	D197E	79.2 ± 2.2	267.1 ± 29.0	214.8 ± 38.2	98.4	7.1 ± 1.5	46.2 ± 29.8	6.2 ± 2.0	4.1
Fold-change^c^			8.3	15.4	11.2	10.1	6.5	35.5	4.8	5.9
A/Fukui/20/2004	A(H3N2)	119E	0.1 ± 0.0	0.1 ± 0.0	0.2 ± 0.1	0.6	1.0 ± 0.4	0.5 ± 0.0	1.4 ± 0.1	1.1
A/Fukui/45/2004	A(H3N2)	E119V	37.0 ± 19.0	34.6 ± 8.9	77.5 ± 9.1	22.9	1.0 ± 0.0	0.5 ± 0.1	1.6 ± 0.2	0.7
Fold-change^c^			370.0	346.0	387.5	38.2	1.0	1	1.1	0.6
A/Mississippi/03/2001	A(H1N1)	275H	0.4 ± 0.0	0.5 ± 0.3	0.7 ± 0.43	0.4	0.3 ± 0.1	0.3 ± 0.1	0.4 ± 0.1	0.4
A/Mississippi/03/2001	A(H1N1)	H275Y	392.7 ± 5.9	272.0 ± 112.6	547.1 ± 47.0	257.3	0.4 ± 0.0	0.4 ± 0.1	0.5 ± 0.1	0.4
Fold-change^c^			981.8	544.0	781.6	643.3	1.3	1.3	1.3	1.0
A/Perth/265/2009	A(H1N1)pdm09	275H	0.2 ± 0.0	0.4 ± 0.2	0.6 ± 0.5	0.3	0.2 ± 0.0	0.2 ± 0.0	0.2 ± 0.1	0.2
A/Perth/261/2009	A(H1N1)pdm09	H275Y	191.3 ± 38.6	131.9 ± 25.2	227.8 ± 8.9	154.9	0.3 ± 0.0	0.2 ± 0.0	0.4 ± 0.2	0.5
Fold-change^c^			956.5	329.8	379.7	516.3	1.5	1.0	2.0	2.5

a This panel of reference viruses is prepared by the Melbourne, Australia, WHO CC and available through the International Society for Influenza and other Respiratory Virus Diseases Antiviral Group (http://www.isirv.org/site/index.php/reference-panel).

b Amino acid position numbering A subtype and B type specific.

c IC_50_ fold-change of the virus with the substitution compared to the virus of the same type/subtype without the substitution.

**Table 2:** Virus and patient characteristics of 66 viruses showing RI or HRI, tested by WHO CCs.^a^

Virus	*n*	IC_50_ fold-change compared to reference median or mean IC_50_ values^b^				NA-substitution^c^		Patient setting	Antiviral treatment	Immuno-compromised
	
		Oseltamivir	Zanamivir	Peramivir	Laninamivir	Virus isolate	Clinical specimen			
A(H1N1)pdm09; *N* = 2343	18	**207–4010**	1.0–6.5	**157–1672 (13)**	1.0–5.1 (13)	H275Y	H275Y (9)	Community (8) Hospital (2)	Yes, oseltamivir (1) Yes, not specified (1) No (8)	No (5)
	1	3.0	**19**	**14**	5.0	D151D/E	na	na	na	na
	1	0.6	**200**	**234**	**33**	Q136R	136Q	Community	No	No
	1	0.6	**185**	**143**	**42**	Q136 K	na	na	na	na
	1	**124**	9.0	na	na	N295S	N295S	Hospital	na	na
A(H3N2); *N* = 5109	3	**120–454**	1.2–5.7	0.9–3.7	1.3–3.0	E119V (1 mixed)	E119V (3 mixed)^d^	Hospital (1)	Yes, oseltamivir (1) Yes, not specified (1)	Yes (1)
	1	**67,338**	**53**	**474**	5.3	R292 K	R292 K	Hospital	Yes, oseltamivir	Yes
	1	4.6	**17**	na	na	None	na	Community	No	na
	16	**2.4–19**	0.5–7.5	**0.1–14**	0.7–4.8	Not sequenced	Na	Community (2) Hospital (5)	na	na
B/Victoria-lineage; *N* = 1763	2	1.7–2.4	0.3–0.4	**6.9–8.1**	0.6–0.7	E264V	Na	na	na	na
	2	3.6–3.7	0.6–1.2	**210–322**	1.2–1.9	H273Y	na	Community	No	No
	1	1.5	0.9	**7.1**	0.9	G148G/R, K343E^e^	na	na	na	na
	1	2.9	1.6	**6.0**	0.9	Y142H	142Y	Community	na	na
	1	1.1	0.9	**18.7**	0.6	V71A, H439R	V71A, 439H	na	na	na
	1	3.4	**5.30**	na	na	None	na	na	na	na
	9	**1.0–5.4**	0.4 – 3.3	**1.7–11.9**	0.6–3.1	Not sequenced	na	Community (4)	Yes, zanamivir (1)	No (3)
B/Yamagata-lineage; *N* = 2172	1	**5.0**	1.0	na	na	A55T, H296Y	na	Community	na	na
	1	**7.3**	1.0	na	na	P76S, A358T, E392G	na	Community	na	na
	1	3.0	**5.9**	na	na	None	na	Community	No	na
	3	2.6–3.3	**2.1–5.4**	**5.4–5.5 (2)**	1.7–2.5 (2)	Not sequenced	na	Hospital (2)	No (1)	na

a Between brackets the number of viruses for which data was reported if less than the number reported in column ‘*n*’. RI = reduced inhibition; HRI = highly reduced inhibition; na = not available; None = no amino acid substitutions compared to viruses with NI phenotype.

b RI and HRI fold-change values are displayed underlined and in bold typeface.

c Amino acid position numbering is A subtype and B type specific.

d One virus isolate and all three clinical specimens showed 119E/V polymorphism.

e Two other B/Victoria/2/87 lineage viruses with K343E only did not show RI with any of the antivirals.

**Table 3:** Frequency of amino acid substitutions in influenza NAs, submitted to GISAID and NCBI sequence databases, known to occur clinically and cause clinical resistance.^a^

Type/Subtype	Neuraminidase amino acid substitution^b^	No. of sequences containing the substitution (%)^c^	Virus	Home country patient	Specimen collection date	Included in phenotypic analysis^d^
A(N1)	H275Y	22 (2%)	A/Austria/713625/2013	Austria	8–1-2013	Yes (HRI oseltamivir)
			A/Brazil/229/2012	Brazil	20-5-2012	Yes (HRI oseltamivir)
			A/Brisbane/35/2013	Australia	9-4-2013	Yes (HRI oseltamivir)
			A/Costa Rica/6288/2012	Costa Rica	24-6-2013	Yes (HRI oseltamivir)
			A/IIV-Moscow/34/2013	Russian Federation	28-1-2013	Yes (HRI oseltamivir)
			A/India/P131027/2013	India	27-1-2013	No
			A/Laos/1650/2012	Lao People’s Democratic Republic	27-8-2012	No
			A/Nepal/00854/2012	Nepal	30-9-2012	Yes (HRI oseltamivir)
			A/Netherlands/492/2012	The Netherlands	14-8-2012	Yes (HRI oseltamivir)
			A/Netherlands/507/2012	The Netherlands	17-8-2012	Yes (HRI oseltamivir)
			A/Parana/409/2012	Brazil	22-6-2012	No
			A/Pennsylvania/01/2013	United States of America	18-1-2013	Yes (HRI oseltamivir)
			A/Perth/298/2012	Australia	24-12-2012	Yes (HRI oseltamivir)
			A/Rio Grande Do Sul/687/2012	Brazil	27-6-2012	No
			A/Santa Catarine/229/2012	Brazil	20-5-2012	No
			A/St. Petersburg/151/2013	Russian Federation	20-3-2013	Yes (HRI oseltamivir)
			A/Tennessee/03/2013	United States of sAmerica	26-3-2013	Yes (HRI oseltamivir)
			A/Uppsala/4/2013	Sweden	1-4-2013	No
			A/Washington/24/2012	United States of America	17-6-2012	Yes (HRI oseltamivir)
			A/Wyoming/31/2012	United States of America	26-12-2012	Yes (HRI oseltamivir)
			A/Yokosuka/10/2013	Japan	29-1-2013	Yes (HRI oseltamivir)
			A/Yokosuka/11/2013	Japan	4-2-2013	Yes (HRI oseltamivir)

a As listed in the table on the WHO website, available at: http://www.who.int/influenza/gisrs_laboratory/antiviral_susceptibility/nai_overview/en/; accessed 9 May 2014.

b Amino acid position numbering is N1 specific.

c Percentage based on the number of sequences in the final data set after curation – see Supplementary Table 2.

d Yes indicates that the virus was analysed by a WHO CC. HRI = highly reduced inhibition, assessed by *in vitro* assay for the NAI indicate.

**Table 4:** Frequency of amino acid substitutions in influenza NAs, submitted to GISAID and NCBI sequence databases, known to occur clinically but currently of unknown impact, that cause reduced sensitivity *in vitro*.^a^

Type/Subtype	Neuraminidase amino acid substitution^b^	No. of sequences containing the substitution (%)^c^	Virus	Included in phenotypic analysis^d^
A(N1)	D199N	2 (0.2%)	A/Kentucky/02/2013 A/Paraguay/191/2012	Yes (NI) Yes (NI)
	I223R	0		
	N295S	1 (0.1%)	A/Salta/1341/2012	Yes (HRI oseltamivir)
A(N2)	E119V	4 (0.3%)	A/Massachusetts/07/2013 A/Quebec/8118/2013 A/Texas/08/2013 A/Victoria/92/2012	Yes (HRI oseltamivir) No Yes (HRI oseltamivir) Yes (HRI oseltamivir)
	R292K	1 (0.1%)	A/Kagoshima/2/2012	Yes (HRI oseltamivir/peramivir)
	N294S	0		
B^e^	R150K	0		
	D197E	0		
	D197N	1 (0.1%)	B/South Auckland/8/2012	Yes (NI)
	I221T	1 (0.1%)^f^	B/Iowa/05/2013	No
	N294S	0		
	G407S	0		

a As listed in the table on the WHO website, available at: http://www.who.int/influenza/gisrs_laboratory/antiviral_susceptibility/nai_overview/en/; accessed 9 May 2014.

b Amino acid position numbering is A subtype and B type specific.

c Percentage based on the number of sequences in the final data set after curation – see Supplementary Table 2.

d Yes indicates that the virus was analysed by a WHO CC. NI = normal inhibition; HRI = highly reduced inhibition, assessed by *in vitro* assay for the NAIs indicated.

e The H273Y substitution found in the WHO CC data was not included here, because it did not fulfil the requirements for screening: a new substitution should be present in the clinical specimen and more than a single occurrence if in a patient who has not been treated with a NAI.

f A virus containing an I221N substitution was also present but in phenotypic analyses it showed NI by all NAIs.
