# Supplementary material for: Global update on the susceptibility of human influenza viruses to neuraminidase inhibitors, 2012–2013
Source: Antiviral Res. Author manuscript; Available in PMC 2022 Feb 17. (PMC8851378; doi:10.1016/j.antiviral.2014.07.001)
Supplement: Supp 1 [file NIHMS1774595-supplement-Supp_1.pdf]

Supplementary Table 3. List of submitting and originating laboratories of the sequences retrieved from the GISAID sequence database.

**Submitting laboratory sequence**<br>Originating laboratory virus or clinical specimen	**Number of sequences**
**Cantacuzino Institute**	**8**
Cantacuzino Institute	7
Unknown	1
**Centers for Disease Control and Prevention, Atlanta, USA**	**1137**
ADImmune Corporation	4
ADPH Bureau of Clinical Laboratories	8
Alabama State Laboratory	1
Alaska State Virology Lab	23
All India Institute of Medical Sciences	15
Arizona Department of Health Services	4
Arkansas Children's Hospital	2
California Department of Health Services	28
Caribbean Epidemiology Center	18
CDC-Kenya	13
CEMIC University Hospital	1
CENETROP	7
Centers for Disease Control and Prevention	5
Central Laboratory of Public Health	9
Central Public Health Laboratory, Ministry of Health	6
Colorado Department of Health Lab	10
Connecticut Department. of Public Health	3
Contiguo a Hospital Rosales	1
Corpus Christi-Nueces County Public Health	1
DC Public Health Lab	3
Delaware Public Health Lab	14
Departamento de Laboratorio de Salud Publica	2
Ethiopian Health and Nutrition Research Institute (EHNRI)	3
Florida Department of Health-Jacksonville	25
Florida Department of Health-Tampa	12
Georgia Public Health Laboratory	12
Government Virus Unit	17
Houston Department of Health and Human Services	4
Illinois Department of Public Health-Chicago	6
Illinois Department of Public Health-Springfield	1
Indiana State Department of Health Laboratories	13
Institut Pasteur de Dakar	4
Institute of Epidemiology and Infectious Diseases AMS of Ukraine	8
Institute of Epidemiology Disease Control and Research (IEDCR) & Bangladesh National Influenza Centre (NIC)	33
Instituto Adolfo Lutz	6
Instituto Conmemorativo Gorgas de Estudios de la Salud	5
Instituto de Salud Publica de Chile	26
Instituto Nacional de Enfermedades Infecciosas	12
Instituto Nacional de Higiene "Rafael Rangel"	3
Instituto Nacional de Laboratoriosde Salud (INLASA)	3

Submitting laboratory sequence<br>Originating laboratory virus or clinical specimen	Number of sequences
Iowa State Hygienic Laboratory	14
IRSS	6
Ivanovsky Research Institute of Virology RAMS	2
Kansas Department of Health and Environment	8
Kentucky Division of Laboratory Services	15
Laboratorio de Investigacion / Centro de Educacion Medica y Amistad Dominico Japones (CEMADOJA)	7
Laboratorio de Virologia, Direccion de Microbiologia	3
Laboratorio de Virus Respiratorio	1
Laboratorio Nacional de Influenza	5
Laboratorio Nacional de Virologia	3
Louisiana Department of Health and Hospitals	10
Maine Health and Environmental Testing Laboratory	3
Maryland Department of Health and Mental Hygiene	20
Massachusetts Department of Public Health	14
Michigan Department of Community Health	16
Ministry of Health	14
Ministry of Health, NIHRD	2
Minnesota Department of Health	15
Mississippi Public Health Laboratory	9
Missouri Department. of Health & Senior Services	6
Montana Laboratory Services Bureau	10
Montana Public Health Laboratory	2
NAMRU-6	9
National Center for Laboratory and Epidemiology	12
National Influenza Center	3
National Influenza Center French Guiana and French Indies	1
National Influenza Lab	16
National Influenza Reference Laboratory	3
National Institute of Health	5
National Institute of Hygiene and Epidemiology	11
National Institute of Infectious Diseases (NIID)	2
National Institute of Virology	14
National Microbiology Laboratory, Health Canada	6
National Public Health Laboratory	2
National Virology Laboratory, Center Microbiological Investigations	1
Nebraska Public Health Lab	11
Nevada State Health Laboratory	5
New Hampshire Public Health Laboratories	10
New Jersey Department of Health & Senior Services	16
New Mexico Department of Health	17
New York City Department of Health	14
New York State Department of Health	14
North Carolina State Laboratory of Public Health	24
North Dakota Department of Health	10
Ohio Department of Health Laboratories	13
Oklahoma State Department of Health	8
Oregon Public Health Laboratory	5

Submitting laboratory sequence<br>Originating laboratory virus or clinical specimen	Number of sequences
Oswaldo Cruz Foundation - Ministry of Health	12
Pasteur Institut of Côte d'Ivoire	6
Pennsylvania Department of Health	19
Puerto Rico Department of Health	8
Rhode Island Department of Health	7
Russian Academy of Medical Sciences	16
Seattle & King County Public Health Lab	1
South Carolina Department of Health	8
South Dakota Public Health Lab	8
Southern Nevada Public Health Lab	15
Spokane Regional Health District	7
St. Judes Childrens Research Hospital	2
State of Hawaii Department of Health	33
State of Idaho Bureau of Laboratories	14
State Research Center of Virology and Biotechnology Vector	1
Tennessee Department of Health Laboratory-Nashville	6
Texas Childrens Hospital	3
Texas Department of State Health Services, South Texas Laboratory	1
Texas Department of State Health Services-Laboratory Services	17
U.S. Air Force School of Aerospace Medicine	15
U.S. Naval Medical Research Unit No.3	2
Uganda Virus Research Institute (UVRI), National Influenza Center	1
University of the West Indies	2
US NAMRU-6	10
Utah Department of Health	20
Vermont Department of Health Laboratory	7
Virginia Division of Consolidated Laboratories	11
Washington State Public Health Laboratory	10
West Virginia Office of Laboratory Services	5
WHO Chinese National Influenza Center	16
WHO Collaborating Centre for Reference and Research on Influenza	4
WHO National Influenza Centre, National Institute of Medical Research (NIMR)	22
Wisconsin State Laboratory of Hygiene	26
Wyoming Public Health Laboratory	13
Unknown	1
**Health Protection Agency**	**2**
Centre for Infections, Health Protection Agency	2
**Hellenic Pasteur Institute**	**40**
Hellenic Pasteur Institute	40
**Hospital Clínic de Barcelona**	**39**
Hospital Clínic	37
Subdirección General de Epidemiología y Vigilancia de la Salud	2
**Hospital Universitari Vall d'Hebron**	**26**
Hospital Universitari Vall d'Hebron	26
**Institut Pasteur**	**20**
Institut Pasteur	20
**Instituto de Salud Carlos III**	**26**

Submitting laboratory sequence<br>Originating laboratory virus or clinical specimen	Number of sequences
Instituto de Salud Carlos III	3
Servicio de Microbiología Clínica Universidad de Navarra	2
Servicio de Microbiología Complejo Hospitalario de Navarra	6
Servicio de Microbiología Hospital Donostia	1
Servicio de Microbiología Hospital Miguel Servet	4
Servicio de Microbiología Hospital San Pedro	4
Servicio de Microbiología Hospital San Pedro de Alcántara	6
**Laboratoire National de Santé**	**12**
Laboratoire National de Santé	12
**National Centre for Disease Control (NCDC)**	**5**
National Center for Communicable Diseases	5
**National Influenza Center**	**1**
National Center for Communicable Diseases	1
**National Institute for Health and Welfare**	**5**
National Institute for Health and Welfare	5
**National Institute for Medical Research, London, UK**	**1062**
Aristotelian University of Thessaloniki	3
Barcelona, Facultad de Medicina	10
Cantacuzino Institute	16
Centers for Disease Control and Prevention	3
Central Health Laboratory	10
Central Public Health Laboratory	23
Central Veterinary Institute Budapest	11
Centre Pasteur du Cameroun	44
CRR virus Influenza region Sud	16
Erasmus University of Rotterdam	8
Gart Naval General Hospital	5
Government Virus Unit	31
Health Protection Agency	38
Health Protection Inspectorate	22
Hellenic Pasteur Institute	1
Hopital Cantonal Universitaire de Geneves	10
Hopital Charles Nicolle	2
Institut Pasteur	18
Institut Pasteur d'Algerie	6
Institut Pasteur de Dakar	20
Institut Pasteur de Madagascar	46
Institute of Epidemiology and Infectious Diseases AMS of Ukraine	24
Institute of Immunology and Virology Torlak	10
Institute of Public Health	6
Instituto de Salud Carlos III	8
Instituto Nacional de Enfermedades Infecciosas	30
Instituto Nacional de Saude	35
Istanbul University	5
Istituto Superiore di Sanità	29
Ivanovsky Research Institute of Virology RAMS	12
Laboratoire National de Sante	10

**Submitting laboratory sequence**<br>Originating laboratory virus or clinical specimen	**Number of sequences**
Laboratory Directorate	25
Laboratory for Virology, National Institute of Public Health	24
Laboratory of Influenza and ILI	4
Landspitali - University Hospital	11
Lithuanian AIDS Center Laboratory	21
Mater Dei Hospital	7
Ministry of Health of Ukraine	31
National Centre for Disease Control and Public Health	12
National Centre for Preventive Medicine	18
National Centre of Infectious and	12
National Institute of Infectious Diseases (NIID)	1
National Institute of Public Health	14
National Institute of Public Health - National Institute of Hygiene	6
National Public Health Institute of Slovakia	16
National Reference Laboratory	17
National Virus Reference Laboratory	20
NIC	7
Pasteur Institut of Côte d'Ivoire	7
Republic Institute for Health Protection	9
Robert Koch-Institute	25
Sandringham, National Institute for Communicable D	25
Scientific Institute of Public Health	38
State Agency, Infectology Center of Latvia	6
Statens Serum Institut	10
Swedish Institute for Infectious Disease Control	2
Tehran University of Medical Sciences	9
Universidad de Valladolid	17
University of Ghana	22
University of Sarajevo	1
University of Vienna	15
VACSERA	18
WHO Chinese National Influenza Center	4
WHO Collaborating Centre for Reference and Research on Influenza	2
WHO National Influenza Centre	93
Unknown	1
**National Institute of Hygien**	**3**
Institut National d Hygiene, Ministere de la sante sante	3
**National Institute of Infectious Diseases (NIID), Tokyo, Japan**	**329**
Aichi Prefectural Institute of Public Health	6
Akita Research Center for Public Health and Environment	1
Aomori Prefectural Institute of Public Health and Environment	1
Center for Disease Control	19
Center for Public Health and Environment, Hiroshima Prefectural Technology Research Institute	4
Chiba City Institute of Health and Environment	7
Chiba Prefectural Institute of Public Health	1
Ehime Prefecture Institute of Public Health and Environmental Science	3
Fukui Prefectural Institute of Public Health	5

**Submitting laboratory sequence**<br>Originating laboratory virus or clinical specimen	**Number of sequences**
Fukuoka City Institute for Hygiene and the Environment	3
Fukuoka Institute of Public Health and Environmental Sciences	4
Fukushima Prefectural Institute of Public Health	2
Gifu Municipal Institute of Public Health	3
Gifu Prefectural Institute of Health and Environmental Sciences	1
Gunma Prefectural Institute of Public Health and Environmental Sciences	6
Hamamatsu City Health Environment Research Center	1
Hiroshima City Institute of Public Health	2
Hokkaido Institute of Public Health	1
Hyogo Prefectural Institute of Public Health and Consumer Sciences	7
Ibaraki Prefectural Institute of Public Health	2
Ishikawa Prefectural Institute of Public Health and Environmental Science	4
Kagawa Prefectural Research Institute for Environmental Sciences and Public Health	1
Kagoshima Prefectural Institute for Environmental Research and Public Health	3
Kanagawa Prefectural Institute of Public Health	4
Kawasaki City Institute of Public Health	2
Kitakyusyu City Institute of Enviromental Sciences	3
Kobe Institute of Health	5
Kochi Public Health and Sanitation Institute	4
Kumamoto City Environmental Research Center	2
Kumamoto Prefectural Institute of Public Health and Environmental Science	2
Kyoto City Institute of Health and Environmental Sciences	2
Kyoto Prefectural Institute of Public Health and Environment	1
Mie Prefecture Health and Environment Research Institute	6
Ministry of Health	46
Miyazaki Prefectural Institute for Public Health and Environment	1
Nagano City Health Center	1
Nagano Environmental Conservation Research Institute	13
Nara Prefectural Institute for Hygiene and Environment	1
National Center for Communicable Diseases	8
National Institute of Infectious Diseases (NIID)	1
National Public Health Laboratory	8
Niigata City Institute of Public Health and Environment	6
Niigata Prefectural Institute of Public Health and Environmental Sciences	6
Oita Prefectural Institute of Health and Environment	4
Okinawa Prefectural Institute of Health and Environment	7
Osaka City Institute of Public Health and Environmental Sciences	5
Osaka Prefectural Institute of Public Health	6
Pasteur Centre	5
Research Institute for Environmental Sciences and Public Health of Iwate Prefecture	1
Saga Prefectural Institute of Public Health and Pharmaceutical Research	1
Saitama City Institute of Health Science and Research	4
Saitama Institute of Public Health	5
Sakai City Institute of Public Health	5
Sapporo City Institute of Public Health	7
Sendai City Institute of Public Health	3
Shiga Prefectural Institute of Public Health	3

Submitting laboratory sequence<br>Originating laboratory virus or clinical specimen	Number of sequences
Shimane Prefectural Institute of Public Health and Environmental Science	12
Shizuoka City Institute of Environmental Sciences and Public Health	3
Shizuoka Institute of Environment and Hygiene	4
Tochigi Prefectural Institute of Public Health and Environmental Science	2
Tokyo Metropolitan Institute of Public Health	3
Toyama Institute of Health	1
Wakayama City Institute of Public Health	3
Wakayama Prefectural Research Center of Environment and Public Health	5
Yamagata Prefectural Institute of Public Health	6
Yamaguchi Prefectural Institute of Public Health and Environment	8
Yamanashi Institute for Public Health	3
Yokohama City Institute of Health	12
Yokosuka Institute of Public Health	2
**Norwegian Institute of Public Health**	**26**
Aalesund sjukehus	3
Haukeland University Hospital, Dept. of Microbiology	1
Health Forde, Department of Microbiology	1
Innlandet Hospital Trust, Division Lillehammer, Department for Microbiology	1
Molde Hospital, Laboratory for Medical Microbiology	3
Nordlandssykehuset	1
Oslo University Hospital, Ulleval Hospital, Dept. of Microbiology	2
Ostfold Hospital - Fredrikstad, Dept. of Microbiology	1
Sorlandet Sykehus HF, Dept. of Medical Microbiology	2
St. Olavs Hospital HF, Dept. of Medical Microbiology	6
Universitetssykehuset Nord-Norge HF, Avd. mikrobiologi og smittevern	2
Unknown	3
**Other Database Import**	**233**
Unknown	233
**Public Health Laboratory Services Branch, Centre for Health Protection**	**1**
Public Health Laboratory Services Branch, Centre for Health Protection	1
**Statens Serum Institut**	**1**
Statens Serum Institut	1
**Swedish Institute for Infectious Disease Control**	**94**
Unknown	94
**Swedish National Institute for Communicable Disease Control**	**26**
Swedish Institute for Infectious Disease Control	9
Unknown	17
**USAMRU-K**	**7**
US Army Medical Research Unit - Kenya (USAMRU-K), GEIS Human Influenza Program	7
**WHO Collaborating Centre for Reference and Research on Influenza, Melbourne, Australia**	**298**
Auckland Healthcare	2
Auckland Hospital	2
Austin Health	16
Canberra Hospital	8
Canterbury Health Services	11
Clinical Virology Unit, CDIM	1

**Submitting laboratory sequence**<br>Originating laboratory virus or clinical specimen	**Number of sequences**
Disease Investigation Centre Wates (BBVW)	3
Institut Pasteur New Caledonia	1
Institut Penyelidikan Perubatan	9
Institute of Environmental Science & Research	7
Institute of Environmental Science and Research	25
Institute of Medical and Veterinary Science (IMVS)	32
Institute Pasteur du Cambodia	3
John Hunter Hospital	5
John Hunter Hospital, Virology Unit, Clinical Microbiology	4
Laboratorio De Saude Publico	2
Medical Research Institute	6
Melbourne Pathology	4
Monash Medical Centre	16
NAMRU-2, Cambodia	1
National Centre for Scientific Services for Virology and Vector Borne Diseases	5
National Institute for Communicable Disease	4
National Public Health Laboratory	10
Papua New Guinea Institute of Medical Research	5
Pathwest QE II Medical Centre	10
Prince of Wales Hospital	10
Princess Margaret Hospital for Children	1
Queensland Health Scientific Services	32
Research Institute of Tropical Medicine	6
Royal Chidrens Hospital	2
Royal Darwin Hospital	7
Royal Hobart Hospital	9
Royal Melbourne Hospital	1
University Malaya	1
Victorian Infectious Diseases Reference Laboratory	23
Westmead Hospital	2
WHO National Influenza Centre, National Institute of Medical Research (NIMR)	10
Unknown	2
**WHO National Influenza Centre**	**30**
WHO National Influenza Centre	29
Unknown	1
**Grand Total**	**3431**
