# Supplementary material for: Global update on the susceptibility of human influenza viruses to neuraminidase inhibitors, 2012–2013
Source: Antiviral Res. Author manuscript; Available in PMC 2022 Feb 17. (PMC8851378; doi:10.1016/j.antiviral.2014.07.001)
Supplement: Supp 2 [file NIHMS1774595-supplement-Supp_2.pdf]

Supplementary Table 2. Selection and curation of sequence data from GISAID and NCBI-IVR databases for analysis. [a]

Dataset	Public sequence database	Number of sequences by influenza virus subtype/lineage			
		A(H1N1)pdm09	A(H3N2)	B/Yam	B/Vic
No. of sequences available on-line with: • Minimum length: 1407 nucleotides (H1N1pdm09 and H3N2); 1398 nucleotides (B)	GISAID	1000	1624	831	486
	NCBI Influenza Virus Resource	339	566	321[b]	
Final dataset following removal of: • Sequences without a complete coding region • Repeated sequences between and within databases [c,d] • Sequential degenerate nucleotides [e] • A(H3N2)v sequences [f]	GISAID	863	1347	774	447
	NCBI Influenza Virus Resource	220	1	6 [b]	3 [b]
**Total**		**1083**	**1348**	**780**	**450**

[a] Global Initiative on Sharing All Influenza Data (GISAID) at www.gisaid.org and National Center for Biotechnology Information Influenza Virus Resource (NCBI-IVR) at www.ncbi.nlm.nih.gov/genomes/FLU/FLU.html.

[b] In the NCBI-IVR database, lineage information is not present for most influenza B viruses. Lineage designation was performed after generating independent phylogenies based on HA and NA genes.

[c] Sequences repeated between the two databases were downloaded from the GISAID database only.

[d] Sequences repeated within the same database were analysed for redundancy (Jalview software); when the sequences were redundant, one of the sequences was maintained in the dataset; when the sequences were different, all sequences for that virus were removed from the dataset.

[e] Sequences with potential degenerate nucleotides were not removed unless they contained 3 or more sequential degenerate nucleotides (with none or few nucleotides separating them).

[f] Analysis of the GISAID sequence data for A(H3N2) revealed that A(H3N2)v sequences were included, so datasets without A(H3N2)v sequences were constructed and analysed. When removing the A(H3N2)v sequences it was noticed that other sequences from the same country displayed the same NA sequence pattern at certain nucleotide positions (76G; 90G; 118C; 123T; 127G; 150A; numbering is N2 specific). By considering the NA sequence pattern for removal of sequences, a further 20 sequences were removed to produce the dataset analysed here.
