# Supplementary material for: Global update on the susceptibility of human influenza viruses to neuraminidase inhibitors, 2012–2013
Source: Antiviral Res. Author manuscript; Available in PMC 2022 Feb 17. (PMC8851378; doi:10.1016/j.antiviral.2014.07.001)
Supplement: Supp 3 [file NIHMS1774595-supplement-Supp_3.pdf]

Supplementary Table 1. Statistical significance of differences in IC_{50}s between WHO CCs: effect of normalization using fold-change values compared to the median (four WHO CCs) or mean (Tokyo WHO CC) IC_{50} values. [a]

Virus	Antiviral	IC_{50} values	Number of pairwise comparisons	Number with p-value < 0.05 (Dunn's multiple comparison test)	
		P-value (Kruskal-Wallis test)		IC_{50} values [b]	Fold-change values [c]
A(H1N1)pdm09	Oseltamivir	<0.0001	15	11	0
	Zanamivir	<0.0001	15	11	0
	Peramivir	<0.0001	3	2	2
	Laninamivir	<0.0001	3	2	1
A(H3N2)	Oseltamivir	<0.0001	15	13	2
	Zanamivir	<0.0001	15	15	2
	Peramivir	<0.0001	3	2	2
	Laninamivir	<0.0001	3	3	0
B/Victoria lineage	Oseltamivir	<0.0001	15	14	0
	Zanamivir	<0.0001	15	14	0
	Peramivir	<0.0001	3	3	1
	Laninamivir	<0.0001	3	2	0
B/Yamagata lineage	Oseltamivir	<0.0001	15	13	0
	Zanamivir	<0.0001	15	15	0
	Peramivir	<0.0001	3	2	1
	Laninamivir	<0.0001	3	3	0
Total			144	125	11

[a] Before analysis of the datasets, IC_{50} values and fold-change values were log-transformed. Corresponding graphs with all not transformed IC_{50} and fold-change values are displayed in Supplementary Figure 1.

[b] Median significant p-value < 0.0001; range < 0.0001 – 0.0461.

[c] Median significant p-value 0.0026; range < 0.0001 – 0.0312.
