# Supplementary material for: Global update on the susceptibility of human influenza viruses to neuraminidase inhibitors, 2012–2013
Source: Antiviral Res. Author manuscript; Available in PMC 2022 Feb 17. (PMC8851378; doi:10.1016/j.antiviral.2014.07.001)
Supplement: Supp 4 [file NIHMS1774595-supplement-Supp_4.pdf]

Supplementary Figure 1. Raw IC_{50} and IC_{50} fold-change data generated by individual WHO CCs for specific virus subtypes or lineages and neuraminidase inhibitors.

Each of the five WHO CCs, Atlanta, United States; Beijing, China; London, United Kingdom; Melbourne, Australia; and Tokyo, Japan, provided IC_{50} data for all virus isolates tested with specimen collection dates covering week 21/2012 (19/5/2012) through week 20/2013 (19/5/2013), accompanied by the specimen collection date. All WHO CCs tested for the susceptibility to oseltamivir and zanamivir and the WHO CCs in Atlanta, Melbourne and Tokyo for susceptibility for peramivir and laninamivir also. As the London WHO CC changed its equipment halfway through the study period, their data is displayed for the two periods separately; *London pre* and *London post*. The WHO CCs provided the virus subtype- or lineage-specific median (four WHO CCs) or mean (Tokyo WHO CC) IC_{50} values covering the same period. These values were used to calculate IC_{50} fold-change values for assessment of the levels of inhibition of individual viruses. The normalized data (fold-change values) was used to assess the feasibility of performing comparative analysis of pooled IC_{50} data from different laboratories. Paired column-scatter plots by virus subtype or lineage and neuraminidase inhibitor are shown with individual WHO CCs indicated on the X-axis: the left graphs display the raw IC_{50} data, and those on the right the IC_{50} fold-change data. The Y-axis of the fold-change data is split into three sections indicating the IC_{50} fold-change range for viruses classified as showing ‘normal inhibition’ (NI), ‘reduced inhibition’ (RI) or ‘highly reduced inhibition’ (HRI), compared to the mean or median IC_{50} for susceptible circulating (NI) viruses from the same A subtype or B lineage, as previously recommended by the WHO-AVWG (WHO 2012). Viruses showing NI are influenza A viruses that have <10-fold increase in IC_{50}, or influenza B viruses with <5-fold increase in IC_{50}. Viruses showing RI are influenza A viruses that have a 10- to 100-fold increase in IC_{50}, or influenza B viruses with a 5- to 50-fold increase in IC_{50}. Viruses showing HRI are influenza A viruses with a >100-fold increase in IC_{50} or influenza B viruses with a >50-fold increase in IC_{50}. The median IC_{50} and IC_{50} fold-change values are indicated with a black horizontal bar. The statistical analysis for significance of differences between data from individual WHO CCs associated with these graphs is shown in Supplementary Table 1.

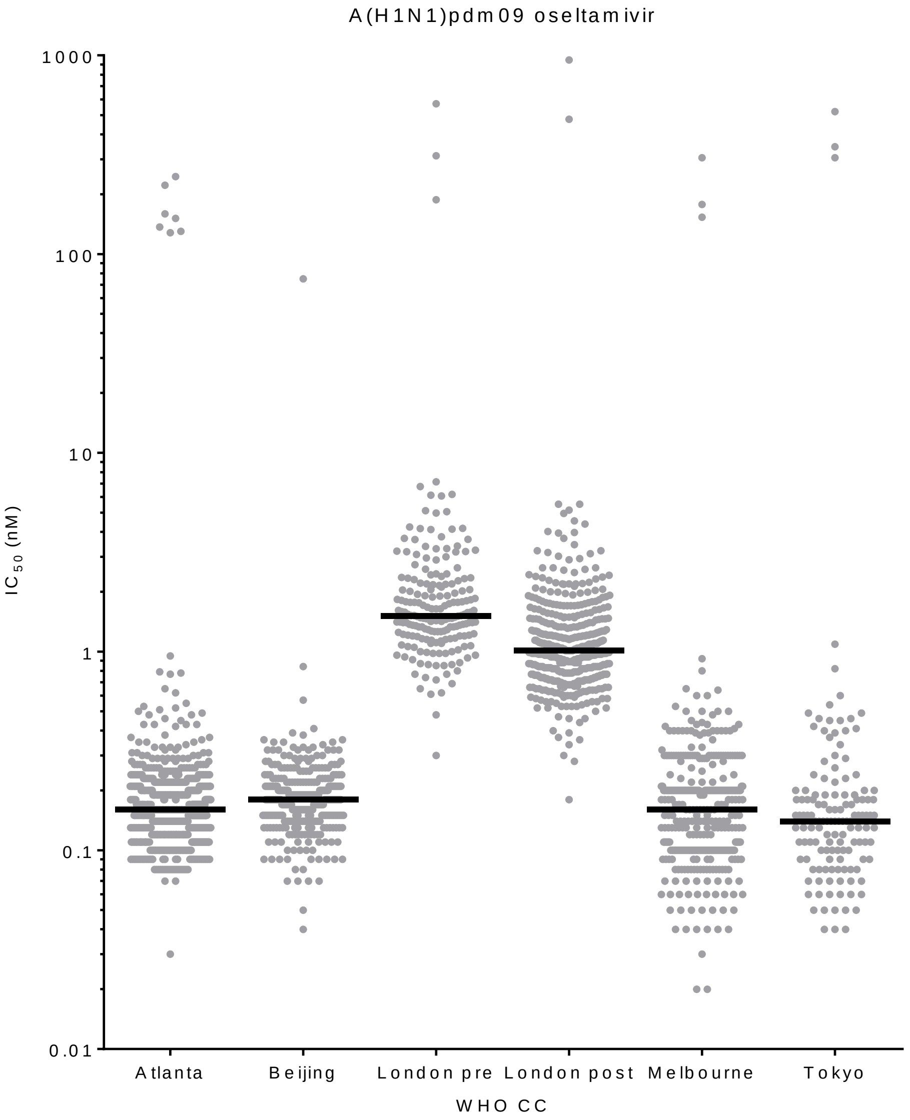
A(H1N1)pdm09 oseltamivir
IC50 (nM)
1000
100
10
1
0.1
0.01
Atlanta
Beijing
London pre
London post
Melbourne
Tokyo
WHO CC

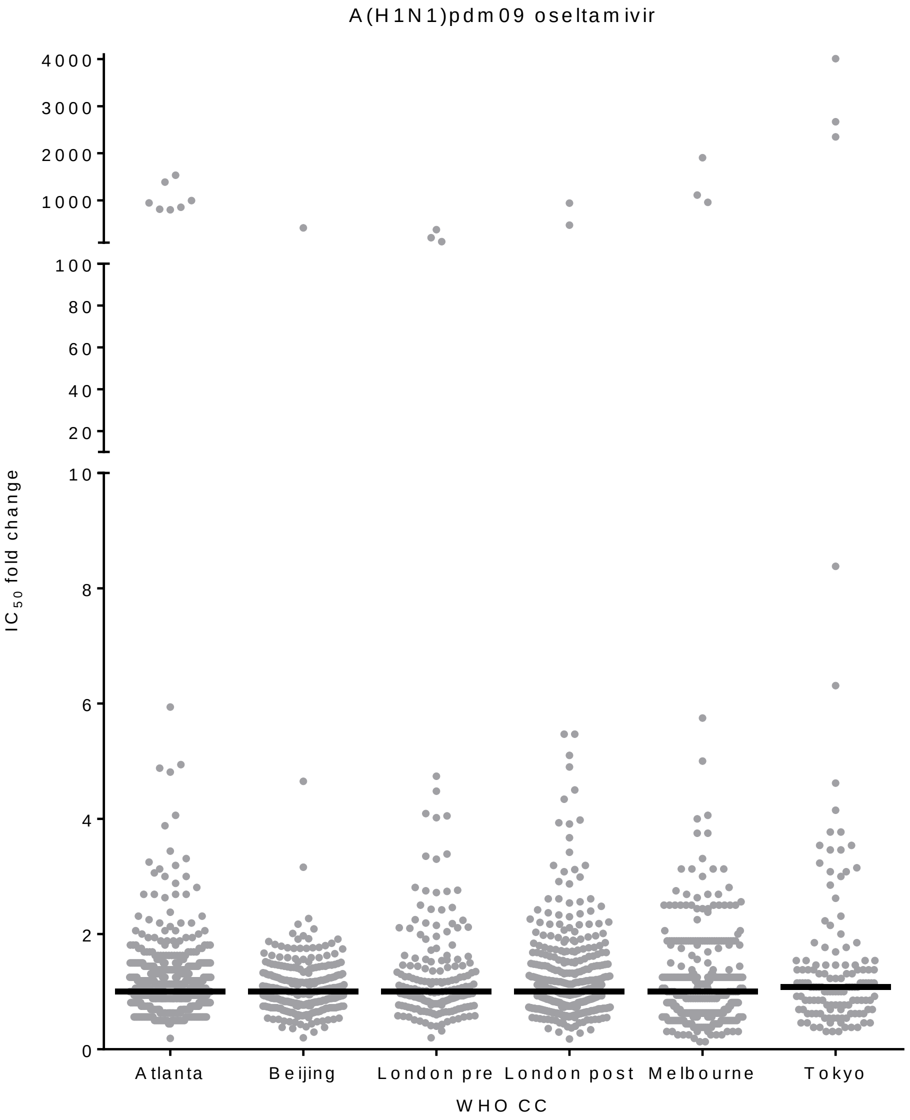
A(H1N1)pdm09 oseltamivir
IC50 fold change
4000
3000
2000
1000
100
80
60
40
20
10
8
6
4
2
0
Atlanta
Beijing
London pre
London post
Melbourne
Tokyo
WHO CC

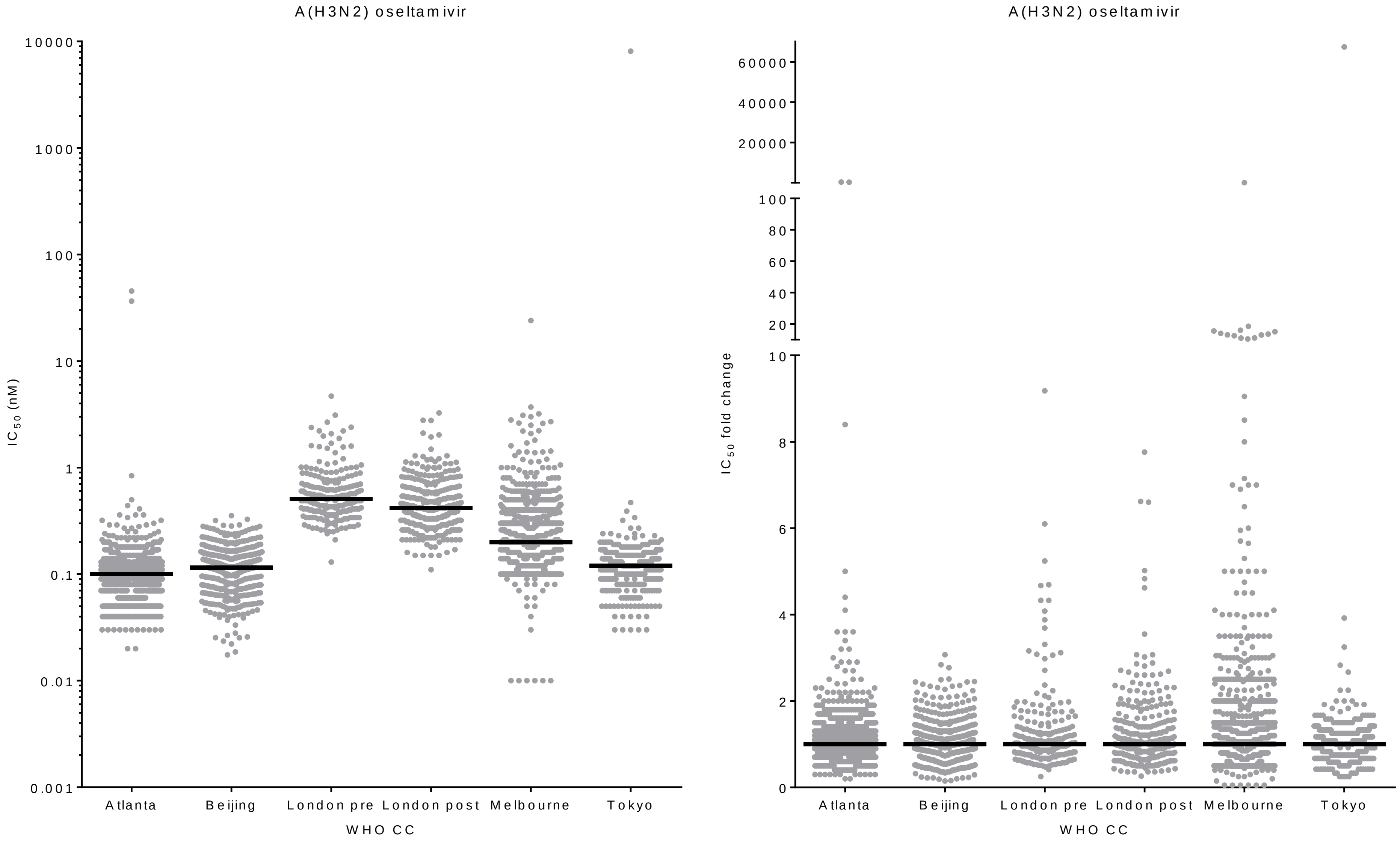

A(H3N2) oseltamivir
10000
1000
100
10
1
0.1
0.01
0.001
IC50 (nM)
Atlanta
Beijing
London pre
London post
Melbourne
Tokyo
WHO CC
A(H3N2) oseltamivir
60000
40000
20000
100
80
60
40
20
10
8
6
4
2
0
IC50 fold change
Atlanta
Beijing
London pre
London post
Melbourne
Tokyo
WHO CC

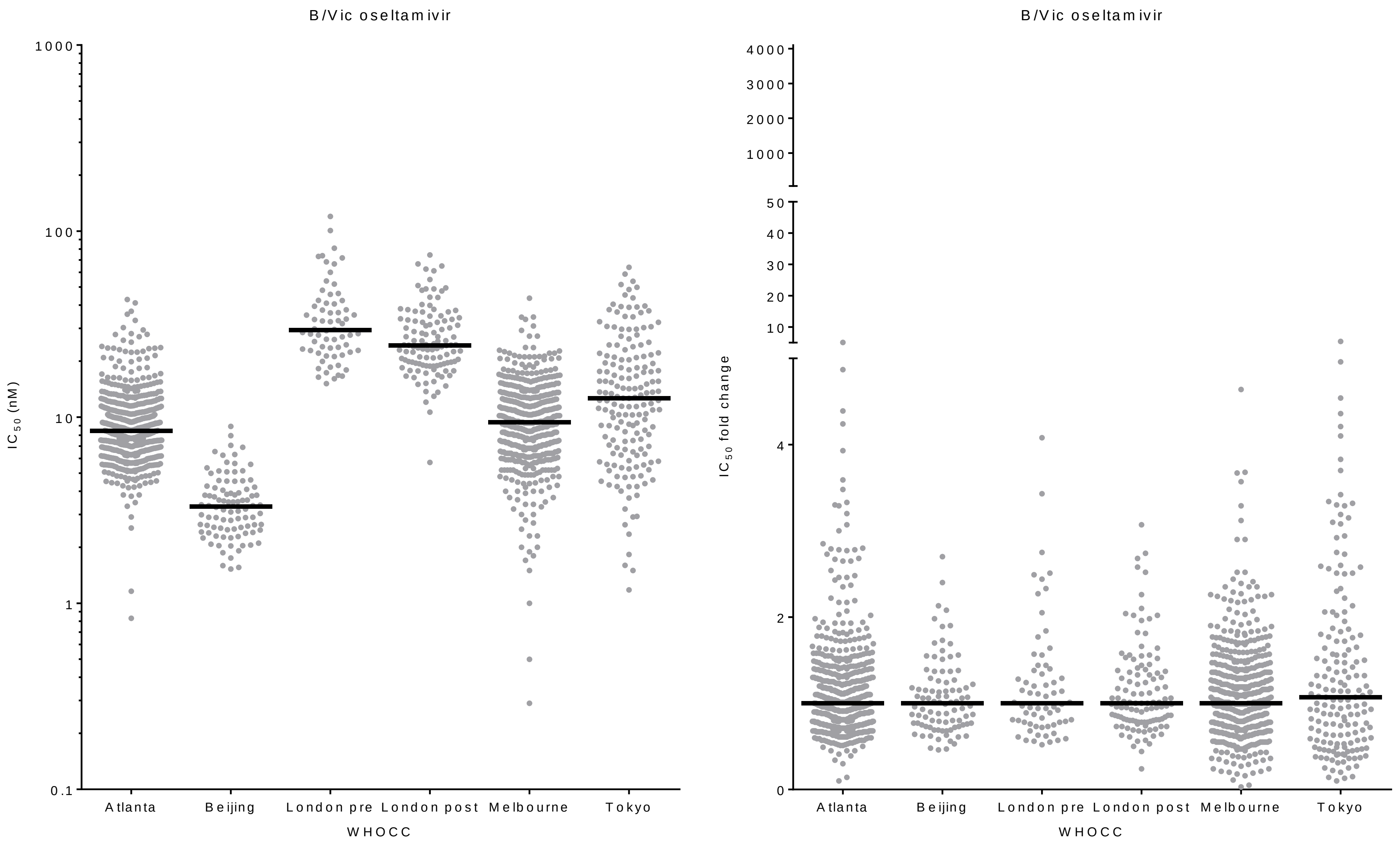

B/Vic oseltamivir
1000
100
10
1
0.1
IC50 (nM)
Atlanta
Beijing
London pre
London post
Melbourne
Tokyo
WHOCC
B/Vic oseltamivir
4000
3000
2000
1000
50
40
30
20
10
4
2
0
IC50 fold change
Atlanta
Beijing
London pre
London post
Melbourne
Tokyo
WHOCC

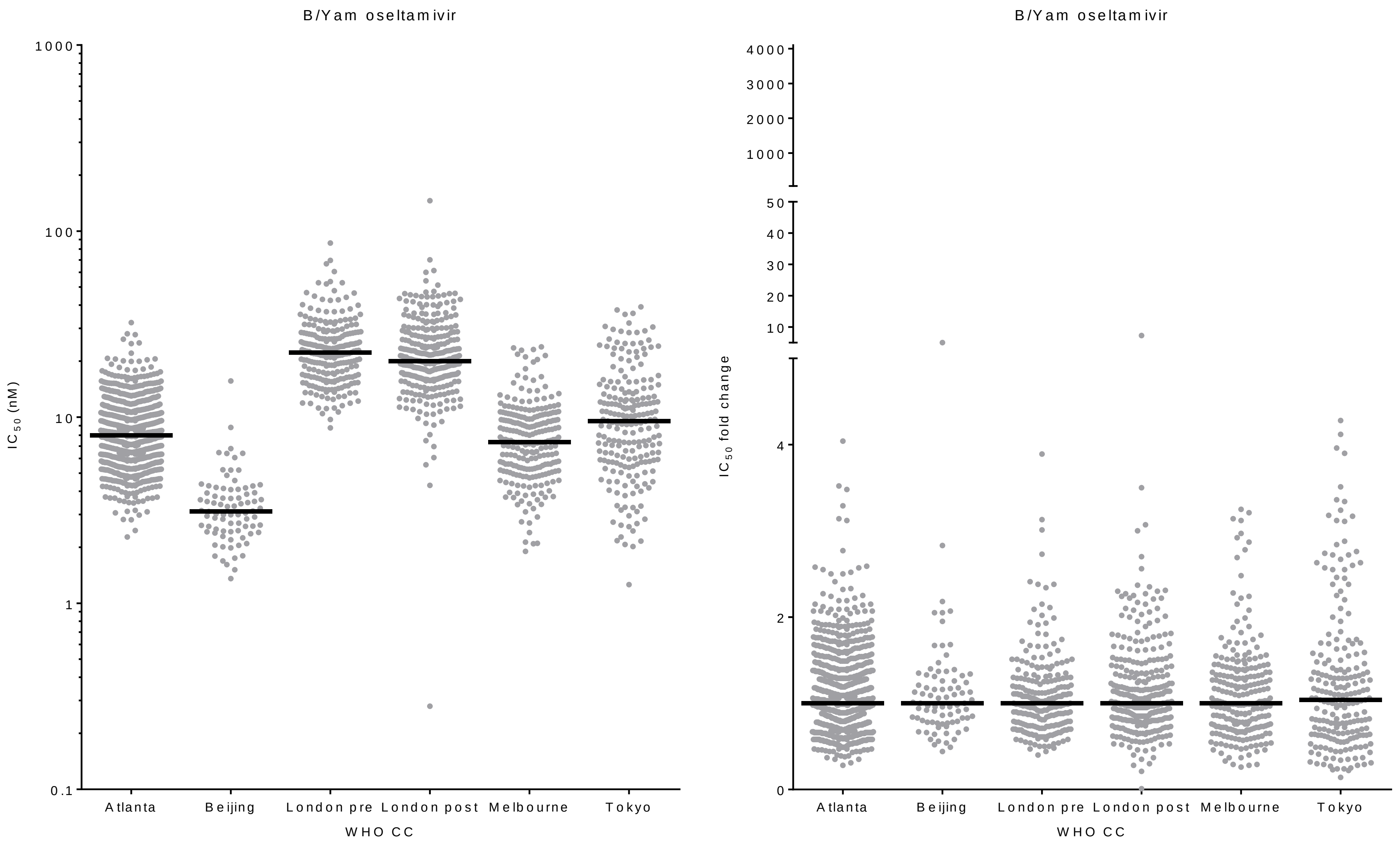
B/Yam oseltamivir
1000
100
10
1
0.1
IC_{50} (nM)
Atlanta
Beijing
London pre
London post
Melbourne
Tokyo
WHO CC
B/Yam oseltamivir
4000
3000
2000
1000
50
40
30
20
10
4
2
0
IC_{50} fold change
Atlanta
Beijing
London pre
London post
Melbourne
Tokyo
WHO CC

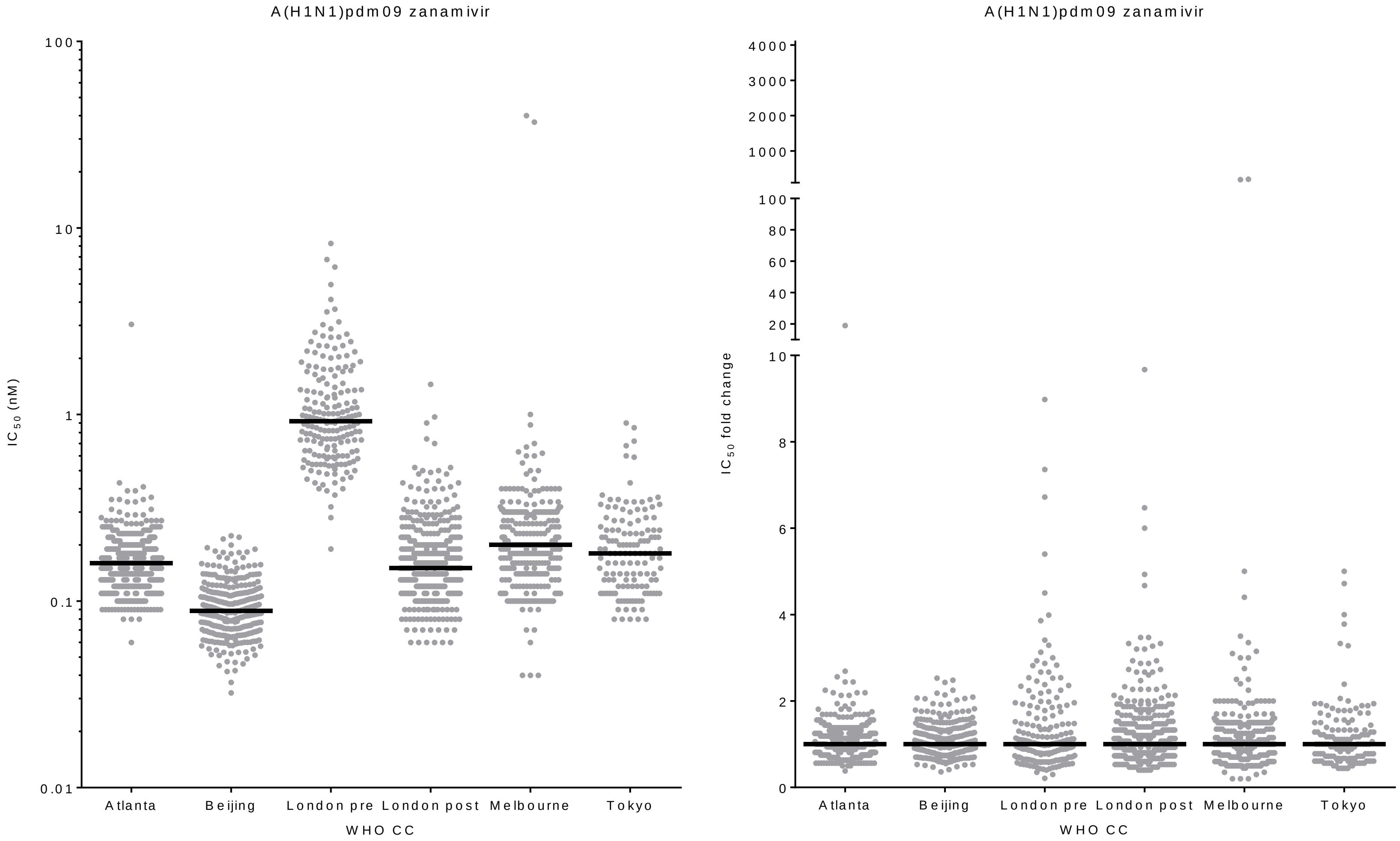

A(H1N1)pdm09 zanamivir
IC50 (nM)
100
10
1
0.1
0.01
Atlanta
Beijing
London pre
London post
Melbourne
Tokyo
WHO CC
A(H1N1)pdm09 zanamivir
IC50 fold change
4000
3000
2000
1000
100
80
60
40
20
10
8
6
4
2
0
Atlanta
Beijing
London pre
London post
Melbourne
Tokyo
WHO CC

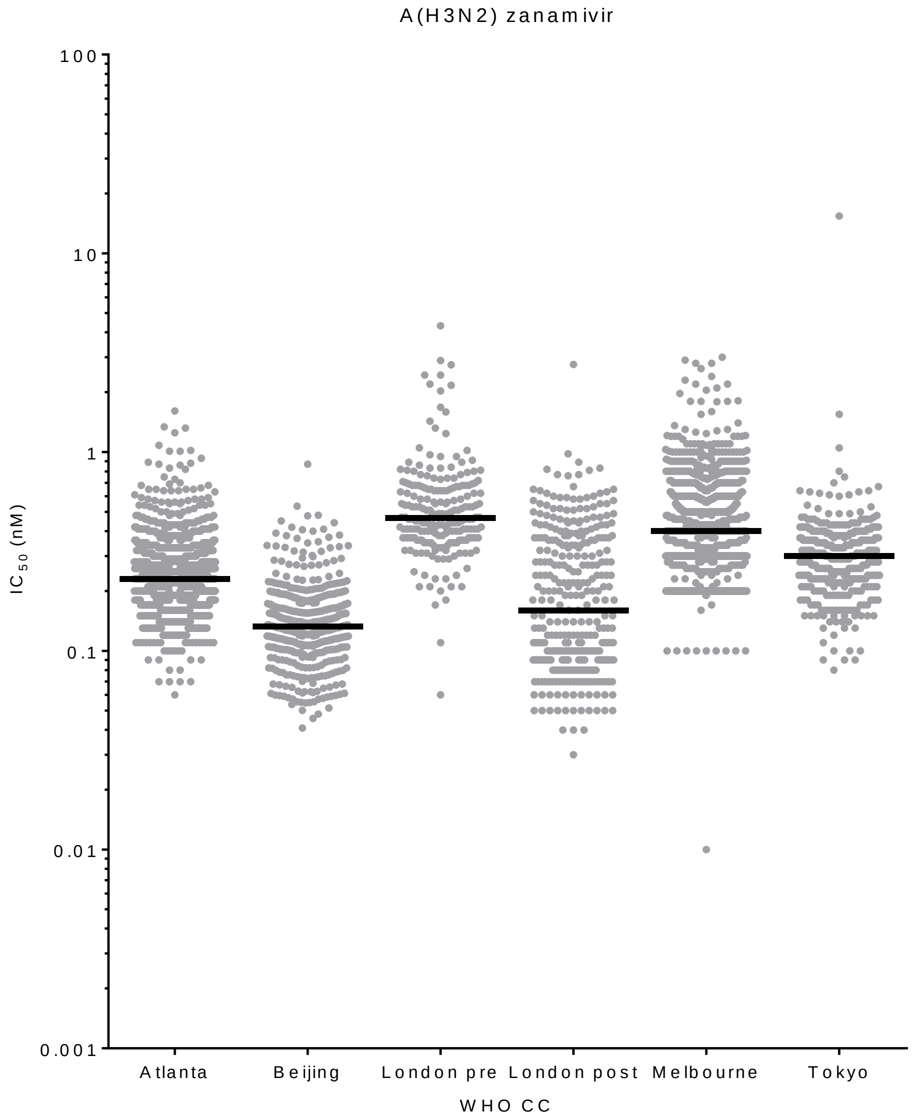
A(H3N2) zanamivir
IC50 (nM)
100
10
1
0.1
0.01
0.001
Atlanta
Beijing
London pre
London post
Melbourne
Tokyo
WHO CC

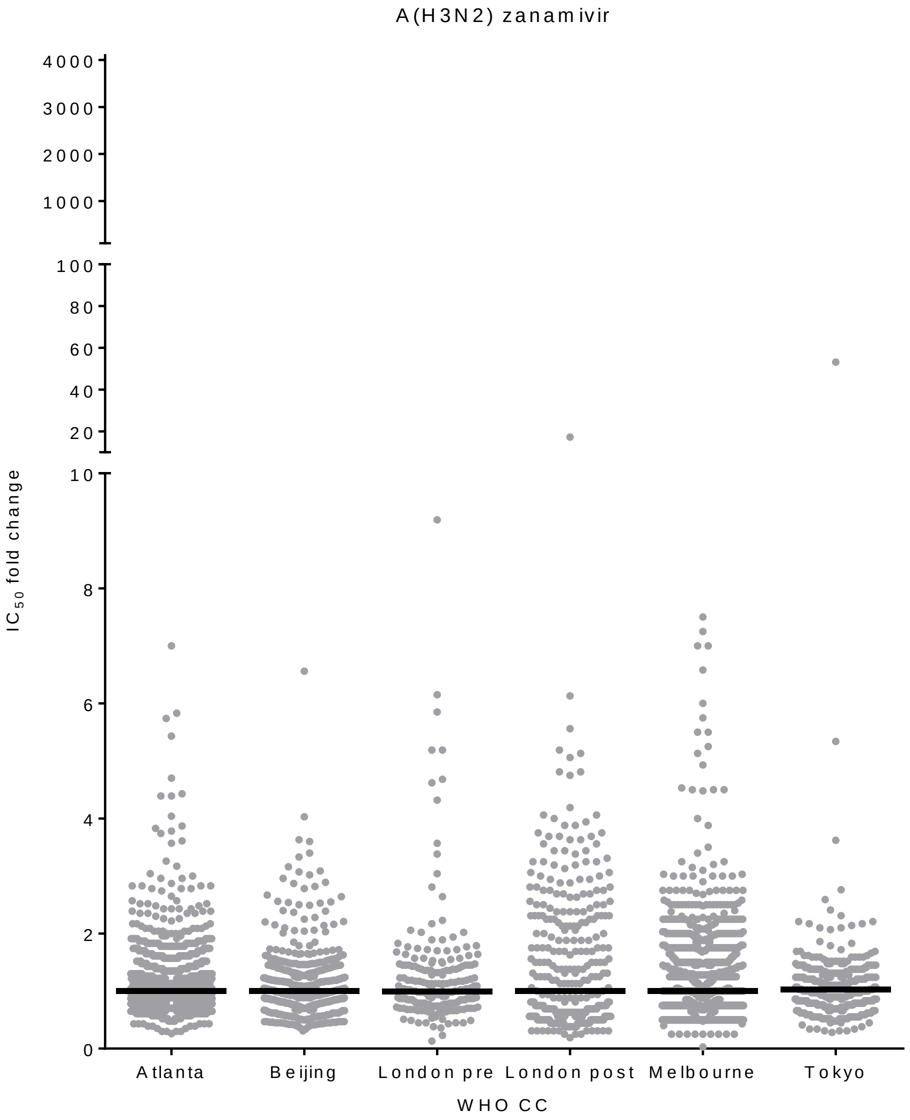
A(H3N2) zanamivir
IC50 fold change
4000
3000
2000
1000
100
80
60
40
20
10
8
6
4
2
0
Atlanta
Beijing
London pre
London post
Melbourne
Tokyo
WHO CC

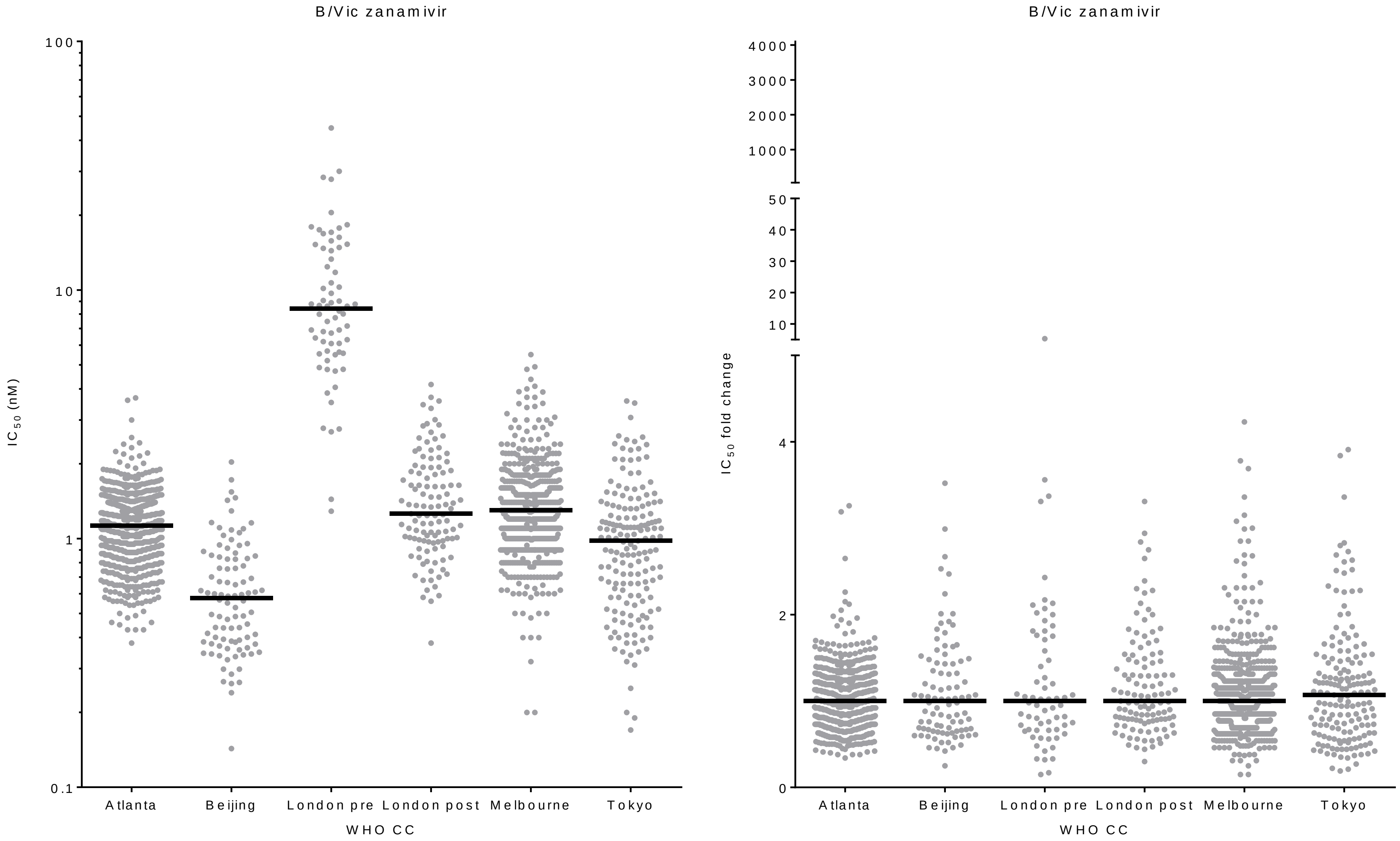
B/Vic zanamivir
IC50 (nM)
100
10
1
0.1
Atlanta
Beijing
London pre
London post
Melbourne
Tokyo
WHO CC
B/Vic zanamivir
IC50 fold change
4000
3000
2000
1000
50
40
30
20
10
4
2
0
Atlanta
Beijing
London pre
London post
Melbourne
Tokyo
WHO CC

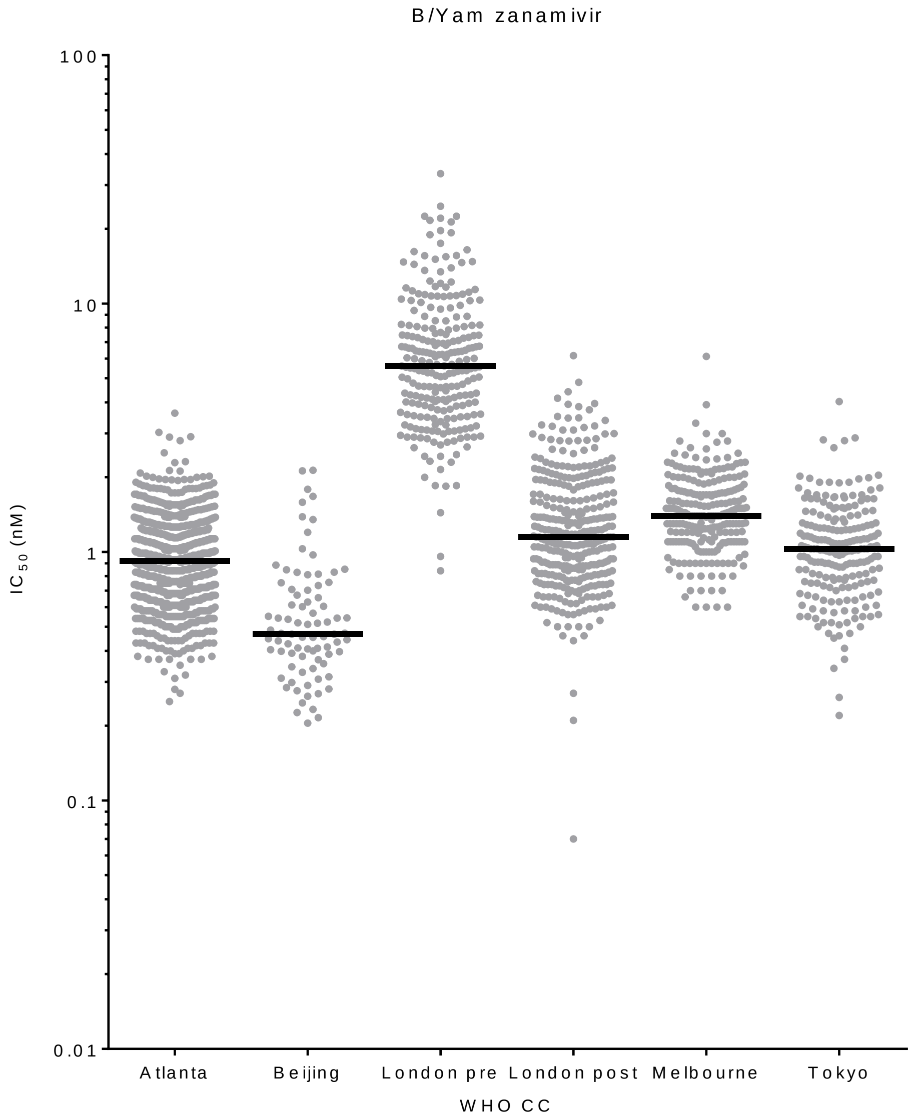
B/Yam zanamivir
100
10
1
0.1
0.01
IC50 (nM)
Atlanta
Beijing
London pre
London post
Melbourne
Tokyo
WHO CC

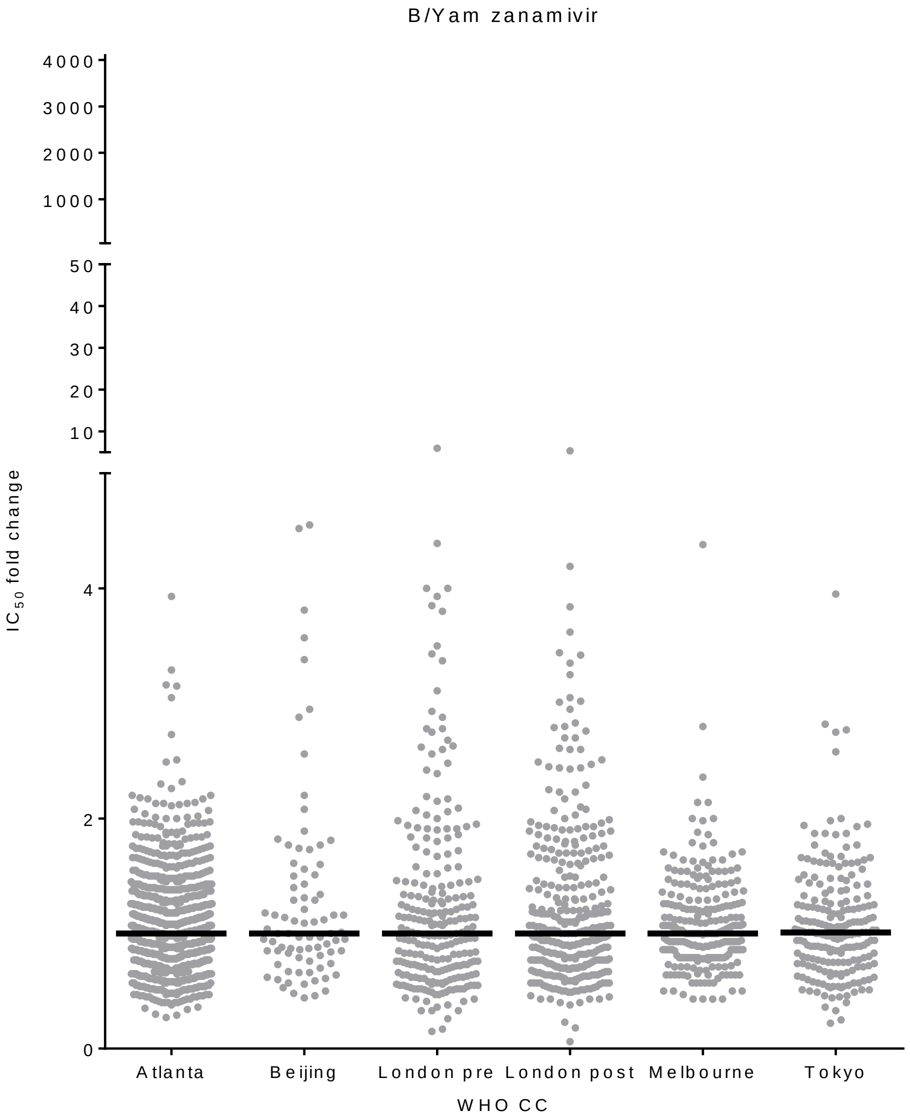
B/Yam zanamivir
4000
3000
2000
1000
50
40
30
20
10
4
2
0
IC50 fold change
Atlanta
Beijing
London pre
London post
Melbourne
Tokyo
WHO CC

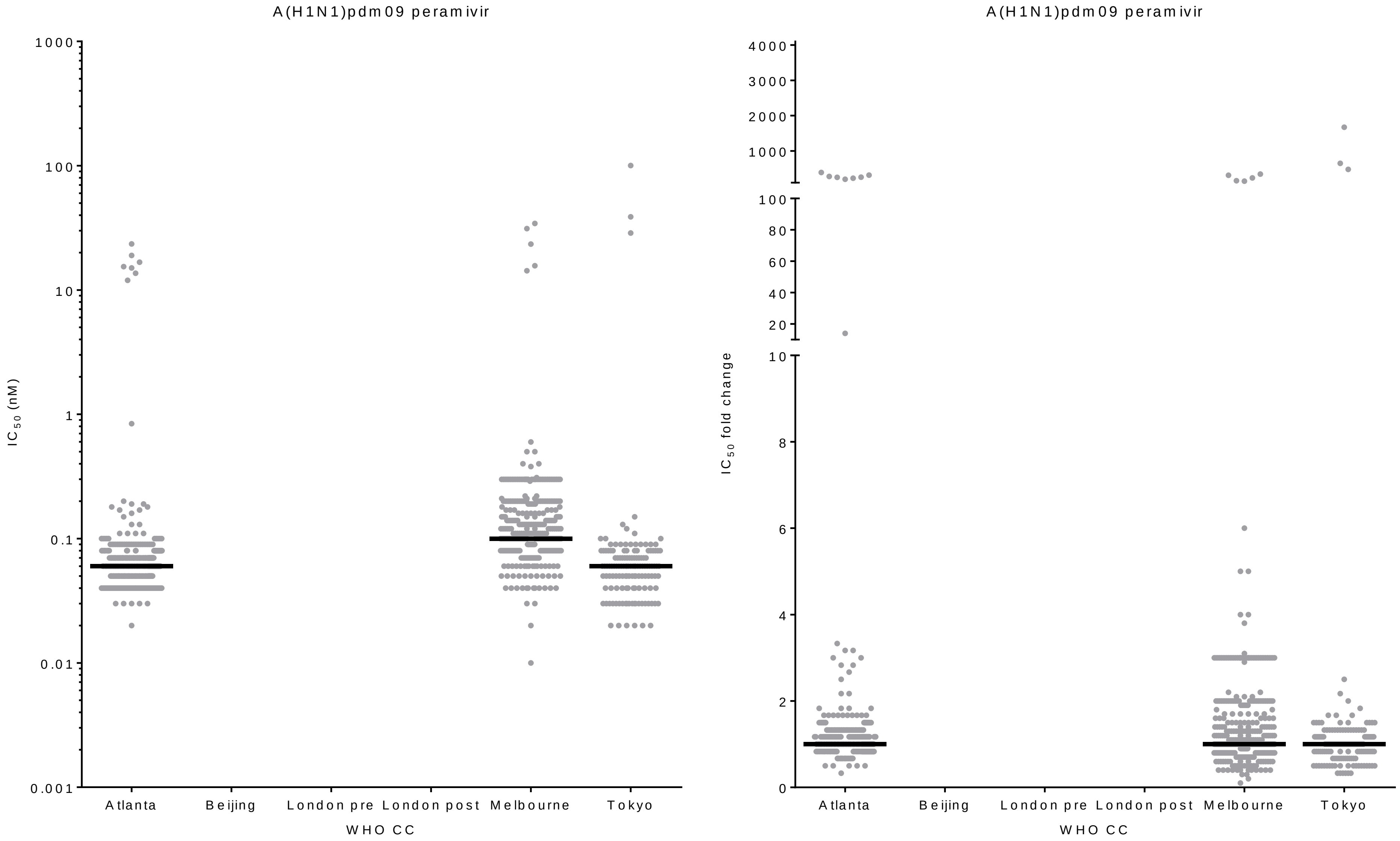
A(H1N1)pdm09 peramivir
IC50 (nM)
1000
100
10
1
0.1
0.01
0.001
Atlanta
Beijing
London pre
London post
Melbourne
Tokyo
WHO CC
A(H1N1)pdm09 peramivir
IC50 fold change
4000
3000
2000
1000
100
80
60
40
20
10
8
6
4
2
0
Atlanta
Beijing
London pre
London post
Melbourne
Tokyo
WHO CC

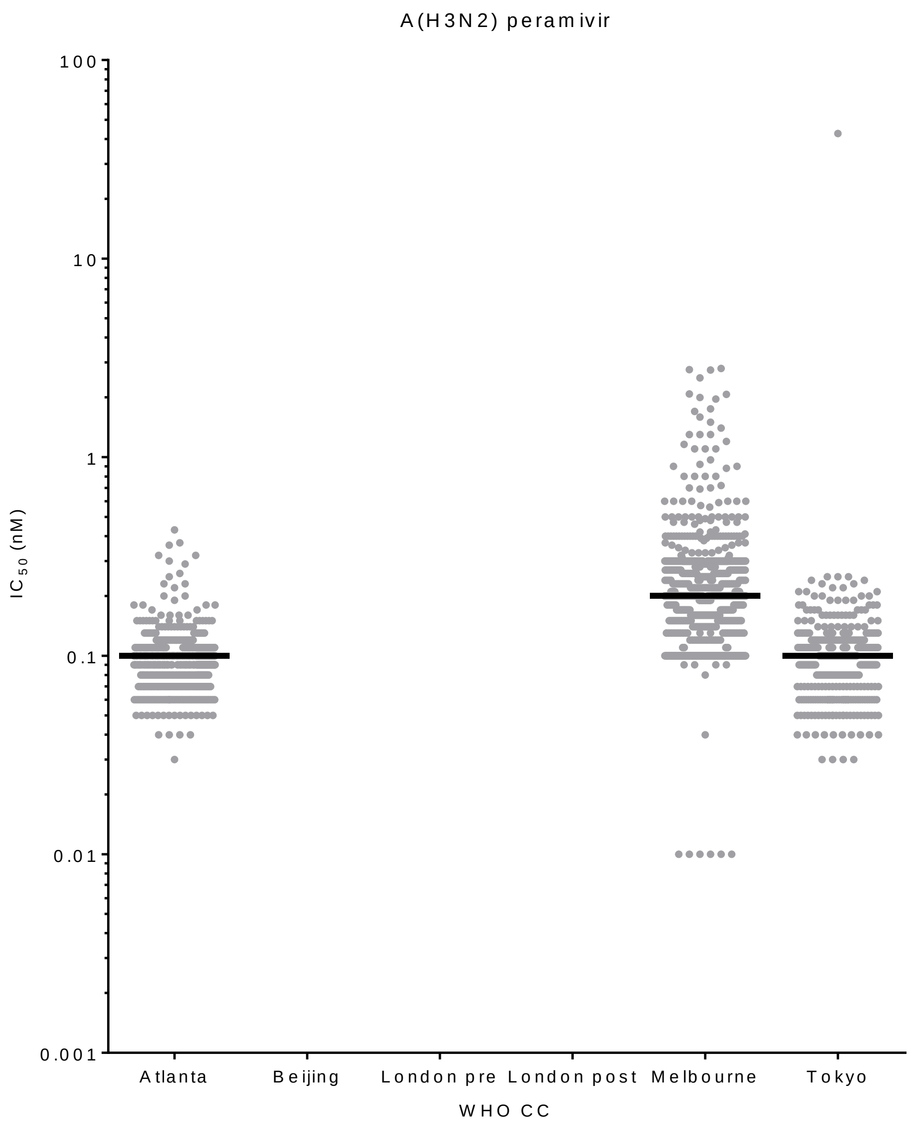

A(H3N2) peramivir
IC50 (nM)
100
10
1
0.1
0.01
0.001
Atlanta
Beijing
London pre
London post
Melbourne
Tokyo
WHO CC

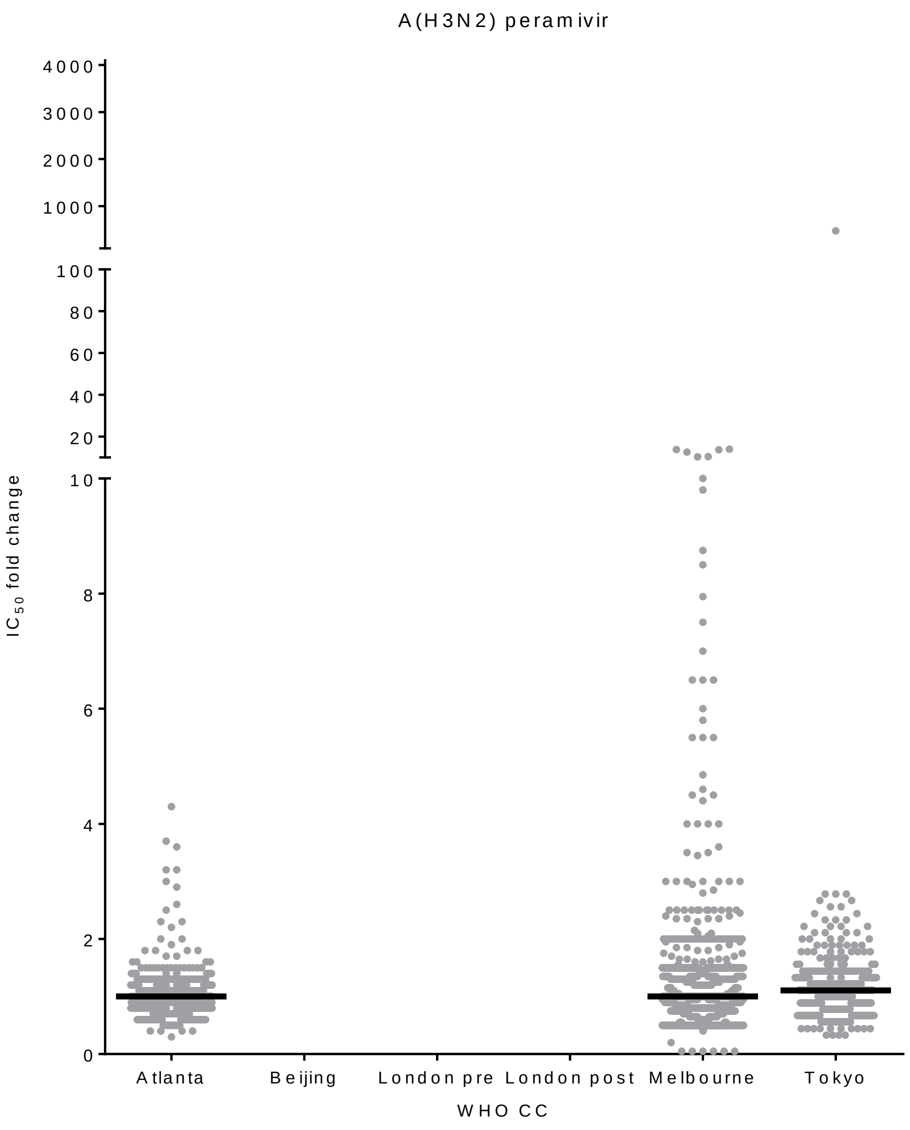

A(H3N2) peramivir
IC50 fold change
4000
3000
2000
1000
100
80
60
40
20
10
8
6
4
2
0
Atlanta
Beijing
London pre
London post
Melbourne
Tokyo
WHO CC

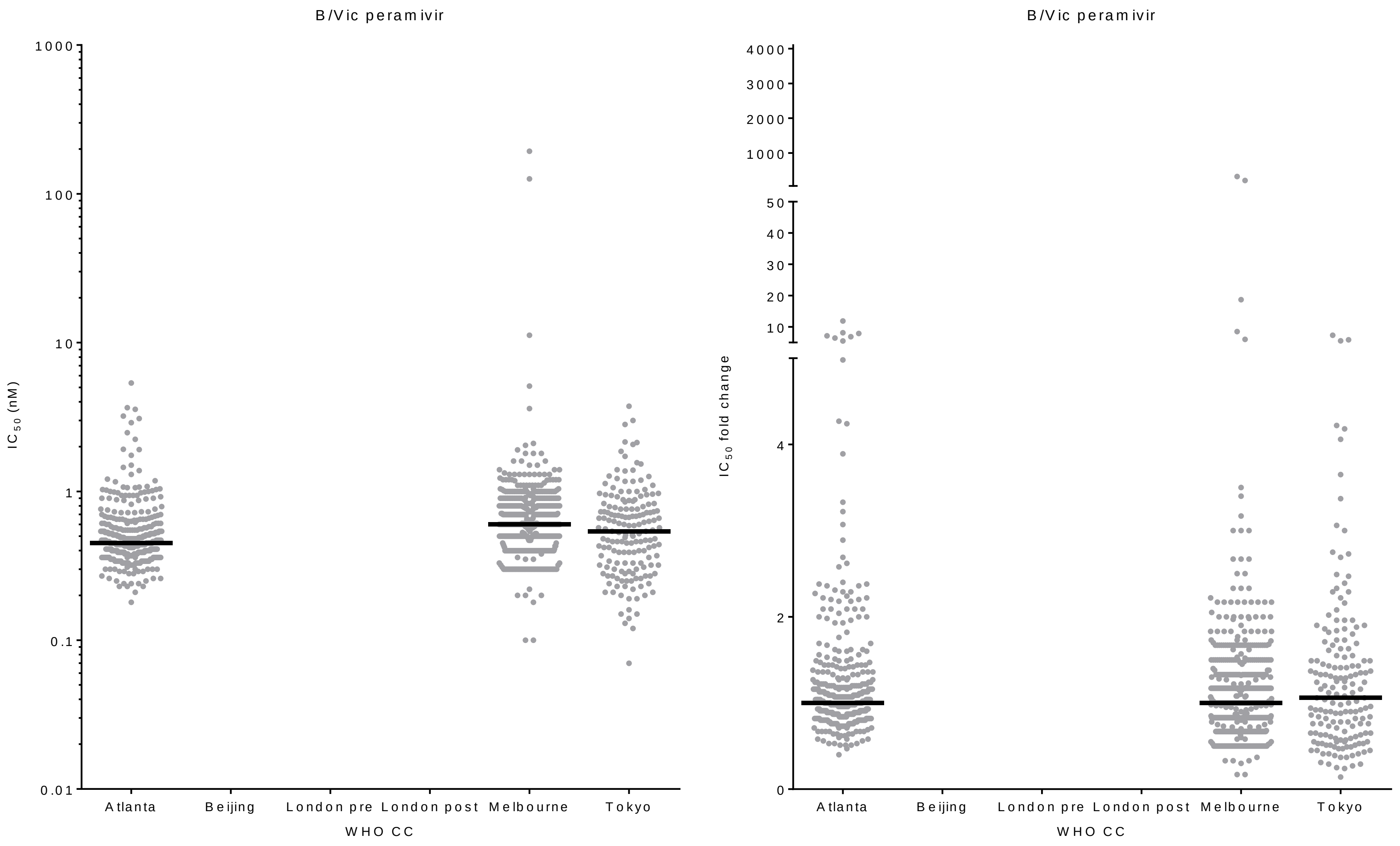
B/Vic peramivir
1000
100
10
1
0.1
0.01
IC50 (nM)
Atlanta
Beijing
London pre
London post
Melbourne
Tokyo
WHO CC
B/Vic peramivir
4000
3000
2000
1000
50
40
30
20
10
4
2
0
IC50 fold change
Atlanta
Beijing
London pre
London post
Melbourne
Tokyo
WHO CC

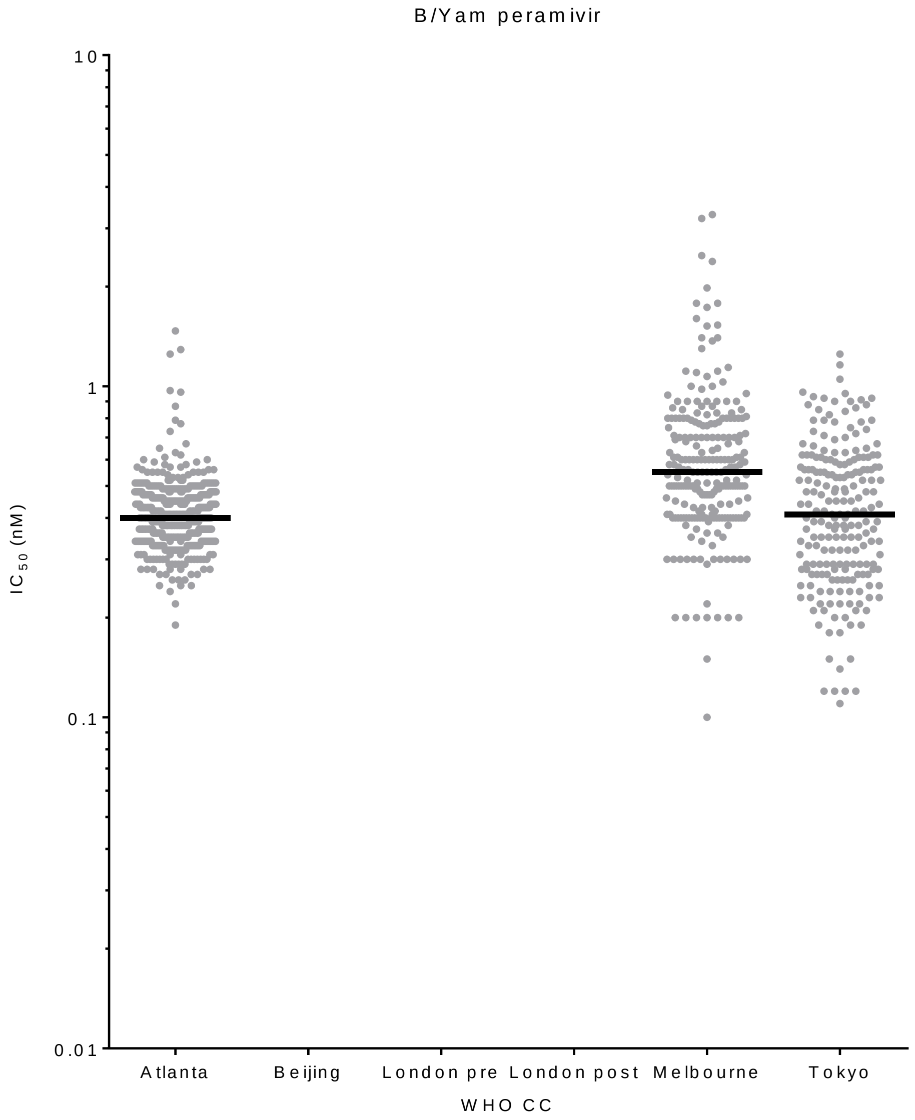
B/Yam peramivir
10
1
0.1
0.01
IC_{50} (nM)
Atlanta
Beijing
London pre
London post
Melbourne
Tokyo
WHO CC

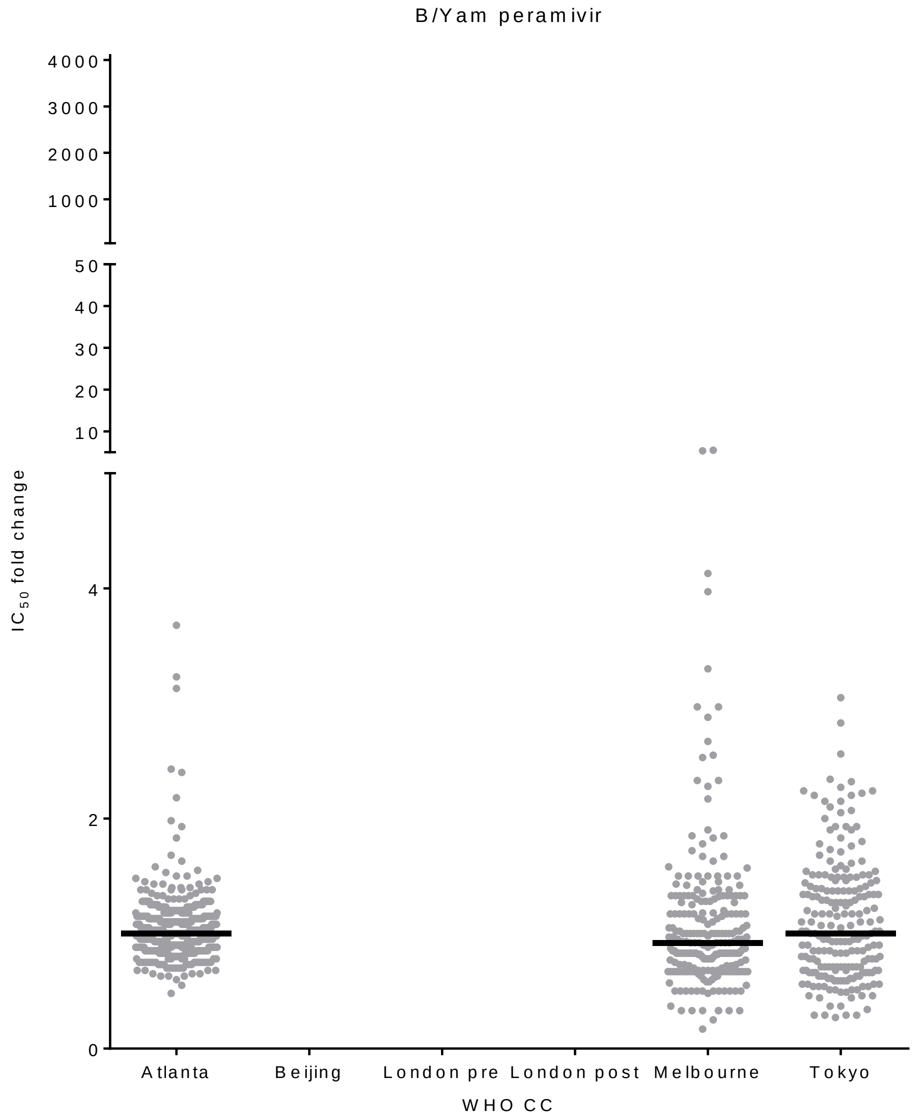
B/Yam peramivir
4000
3000
2000
1000
50
40
30
20
10
4
2
0
IC_{50} fold change
Atlanta
Beijing
London pre
London post
Melbourne
Tokyo
WHO CC

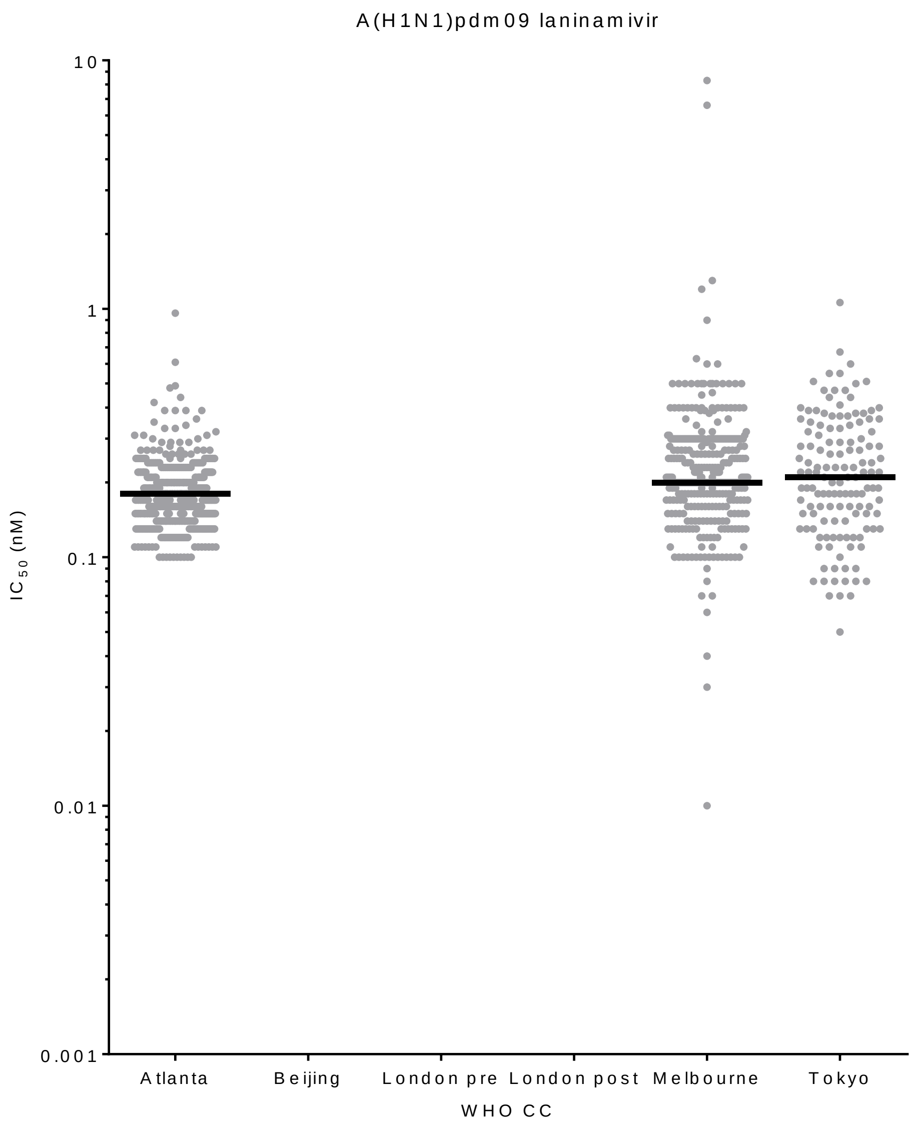
A(H1N1)pdm09 laninamivir
IC50 (nM)
10
1
0.1
0.01
0.001
Atlanta
Beijing
London pre
London post
Melbourne
Tokyo
WHO CC

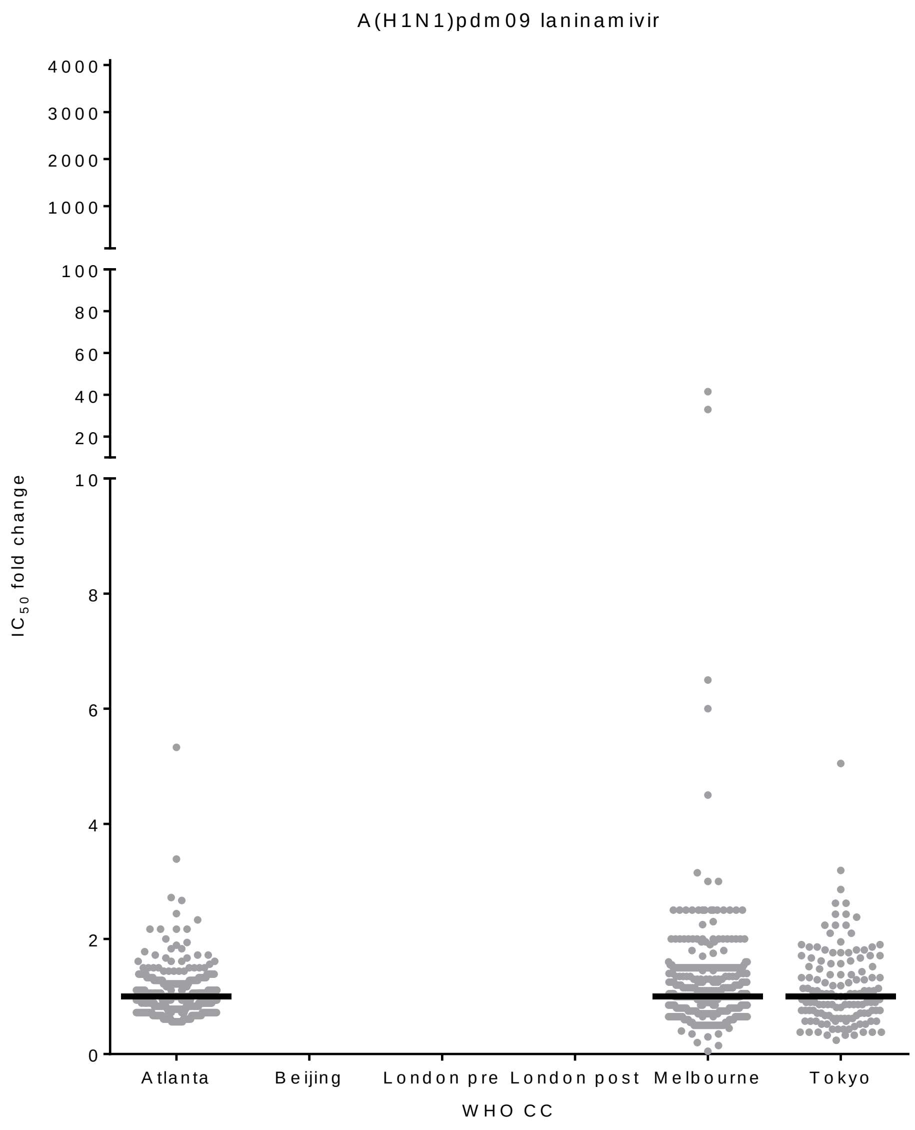
A(H1N1)pdm09 laninamivir
IC50 fold change
4000
3000
2000
1000
100
80
60
40
20
10
8
6
4
2
0
Atlanta
Beijing
London pre
London post
Melbourne
Tokyo
WHO CC

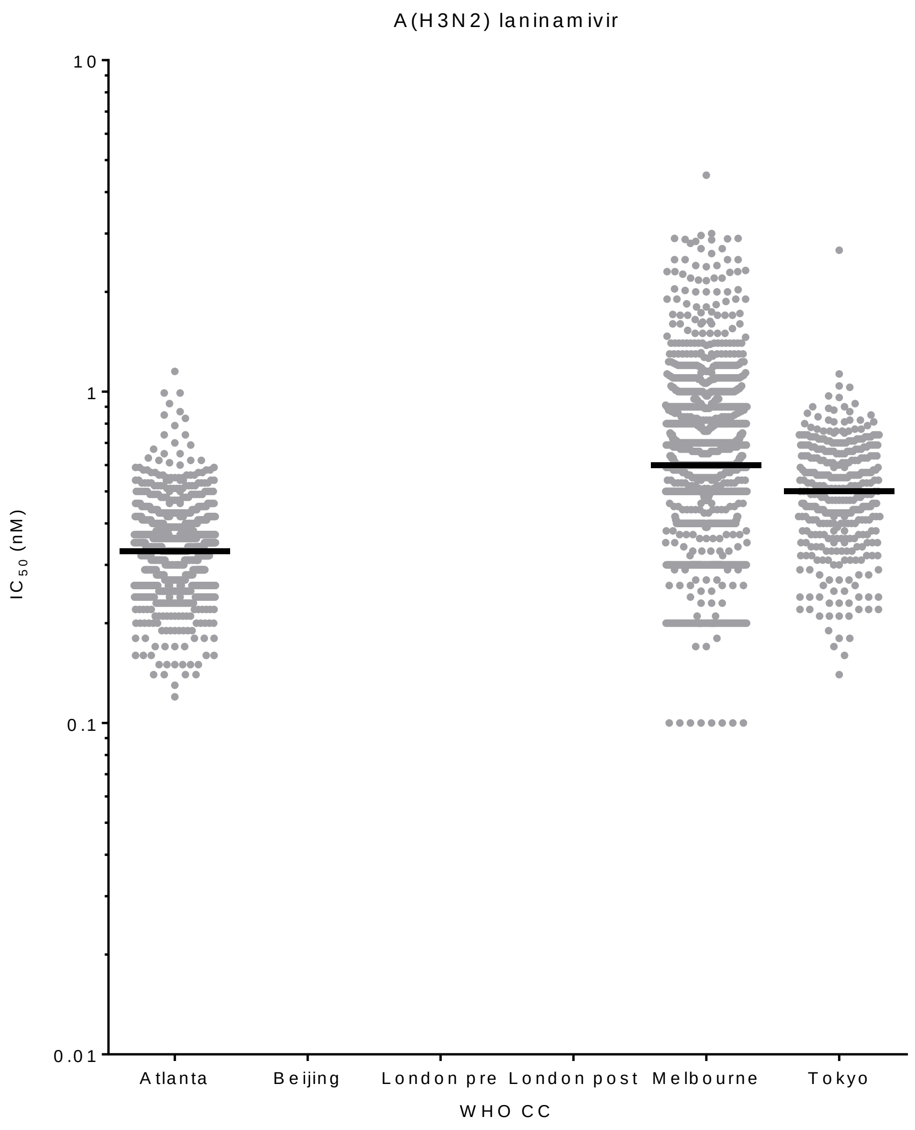
A(H3N2) laninamivir
10
1
0.1
0.01
IC50 (nM)
Atlanta
Beijing
London pre
London post
Melbourne
Tokyo
WHO CC

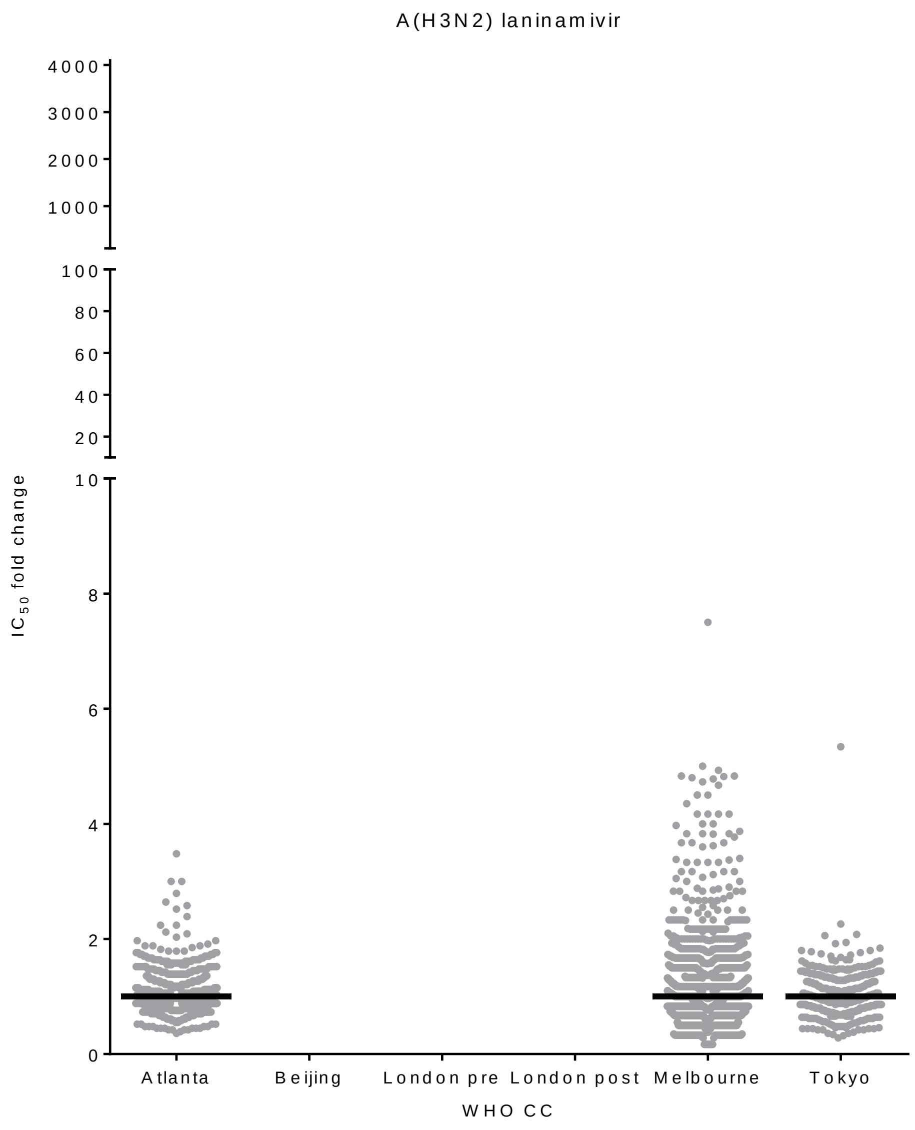
A(H3N2) laninamivir
4000
3000
2000
1000
100
80
60
40
20
10
8
6
4
2
0
IC50 fold change
Atlanta
Beijing
London pre
London post
Melbourne
Tokyo
WHO CC

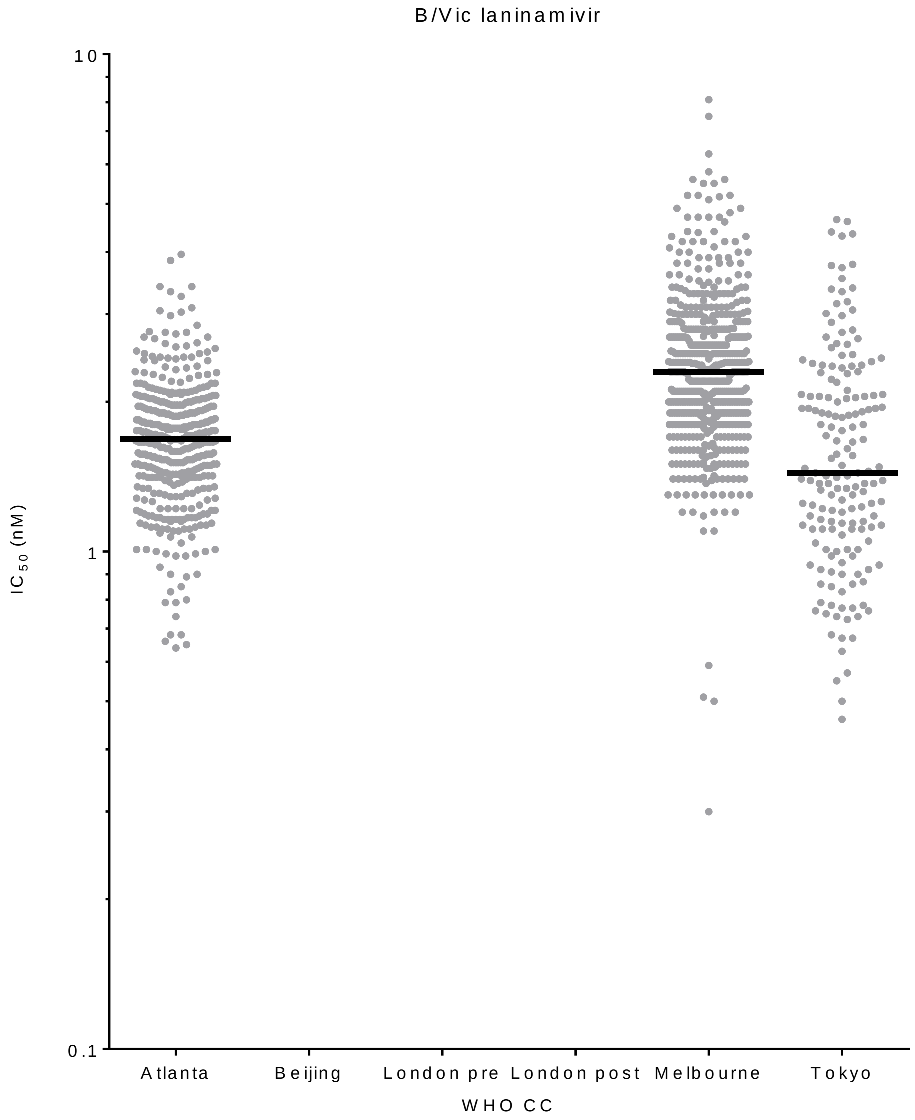
B/Vic laninamivir
10
1
0.1
IC50 (nM)
Atlanta
Beijing
London pre
London post
Melbourne
Tokyo
WHO CC

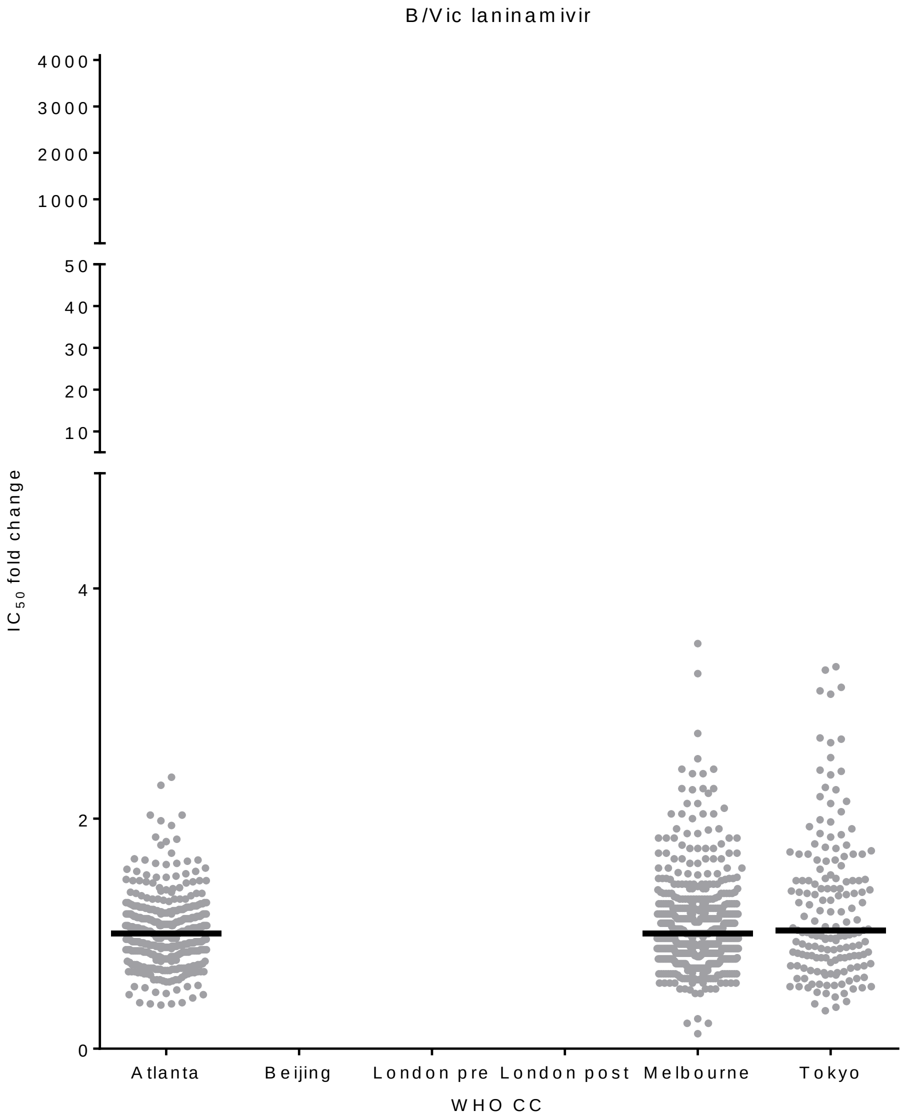
B/Vic laninamivir
4000
3000
2000
1000
50
40
30
20
10
4
2
0
IC50 fold change
Atlanta
Beijing
London pre
London post
Melbourne
Tokyo
WHO CC

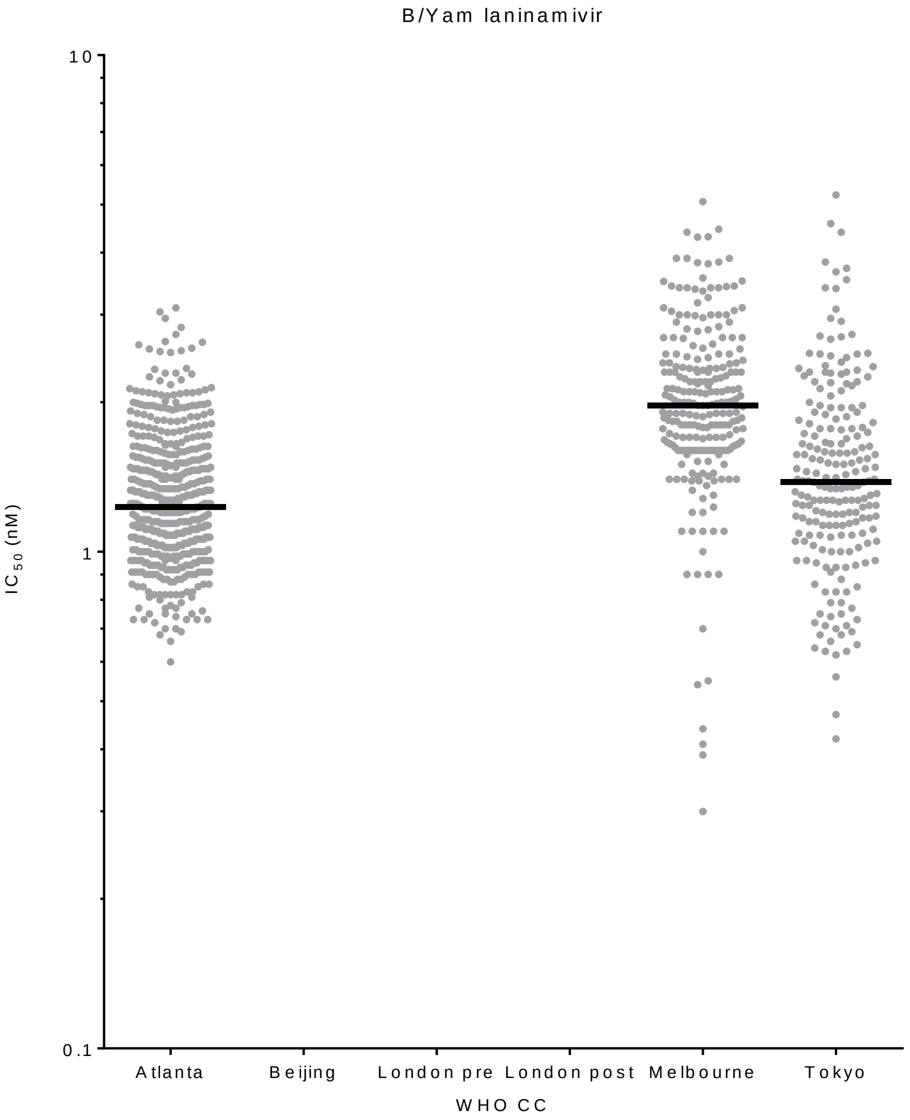
B/Yam laninamivir
10
1
0.1
IC_{50} (nM)
Atlanta
Beijing
London pre
London post
Melbourne
Tokyo
WHO CC

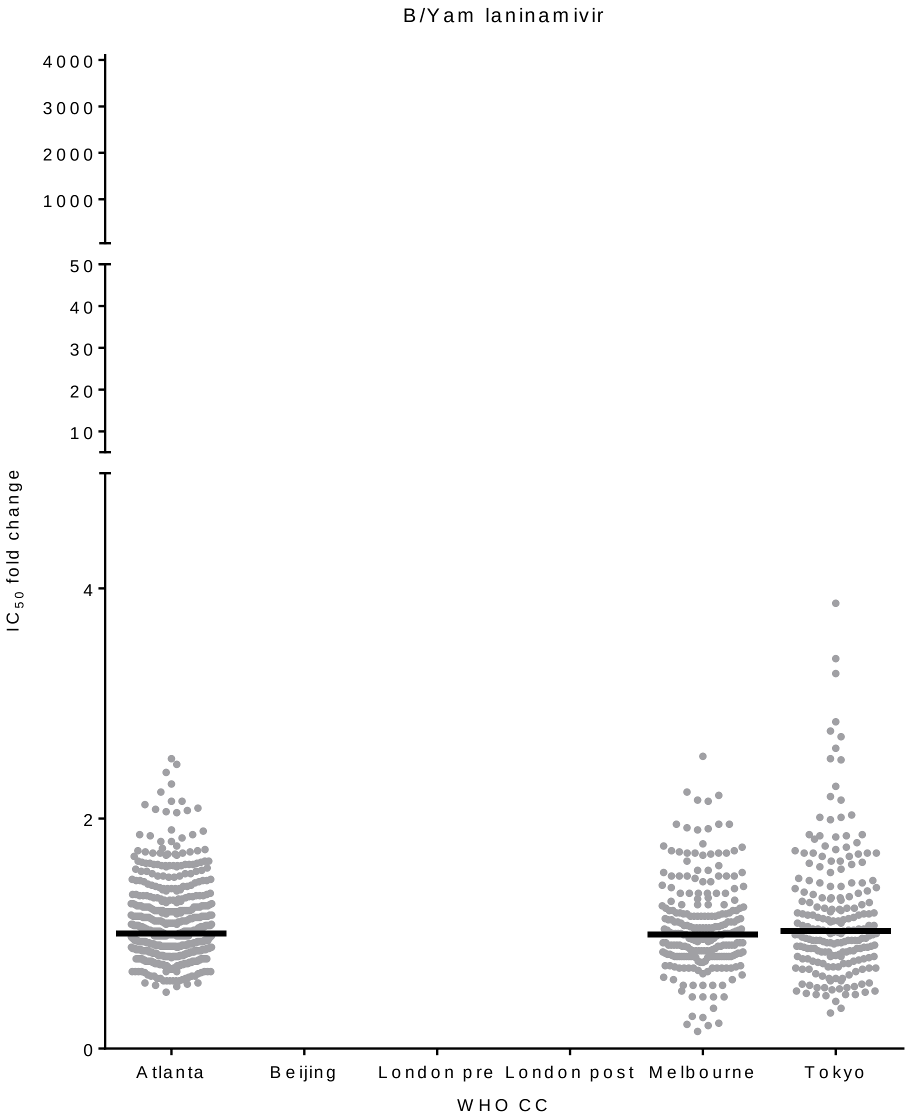
B/Yam laninamivir
4000
3000
2000
1000
50
40
30
20
10
4
2
0
IC_{50} fold change
Atlanta
Beijing
London pre
London post
Melbourne
Tokyo
WHO CC
